# Spillover of sustainable routines from work to private life: application of the Identity and Practice Interdependence Framework

**DOI:** 10.3389/fpsyg.2024.1420701

**Published:** 2024-09-26

**Authors:** Marcia Frezza

**Affiliations:** ^1^Center for Technology and Society (ZTG), Technische Universität Berlin, Berlin, Germany; ^2^Department of Psychology, Health Science Center, Universidade de Fortaleza (UNIFOR), Fortaleza, CE, Brazil

**Keywords:** spillover effects, sustainable routines, pro-environmental behavior, environmental psychology, Identity Process Theory, Social Practice Theory, sustainability management

## Abstract

**Introduction:**

Spillover of sustainable routines and environmentally-responsible behaviors from one setting to another may contribute to achieving essential sustainability goals. Several previous studies on this topic have found few examples of spillover and have focused primarily on individual influences, indicating the need for a better understanding of the factors that have an impact on whether, how, and when spillover occurs. To this end, a novel conceptualization examining the interactions between identity principles and socio-material elements, the Identity and Practice Interdependence Framework, was applied to investigate the occurrence of spillover of sustainable routines from the workplace to home.

**Methods:**

Three focus groups totaling 30 employees of a major Brazilian steel-producing company, interviews in 15 employee homes, and on-site observations of work and private domains provided data that was analyzed qualitatively, using a deductive reflexive thematic approach.

**Results:**

Participants identified 58 changes in home routines related to sustainability as resulting from their experiences at work. With the consistent availability of practice elements (materials, competencies, meanings), learning about, witnessing and performing sustainable routines at work favored satisfactory levels of identity principles (self-efficacy, self-esteem, distinctiveness, continuity). Analysis suggested that seeking the same satisfactions of identity principles outside the workplace led individuals to adapt, change, and/or create more environmentally-responsible routines in their homes.

**Discussion:**

The Identity and Practice Interdependence Framework permitted investigation of the integration of socio-material aspects with the role of individuals in the process of spillover, and indicated some ways they may interact. Both the provision of socio-material components in the first setting and the recognition of more sustainable routines as a feasible path to satisfaction of identity principle needs contribute to individual engagement and persistence in the second setting. Consistent and frequent experiences with more sustainable routines in the first setting or situation may be key to creating this expectation, and therefore to the occurrence of spillover to another setting. The framework complements previous approaches by allowing for a more complex analysis of spillover, which can be used to enrich research on sustainable practices and help promote environmentally-friendly behaviors and sustainable routines, or other desired outcomes, both within organizations and beyond.

## 1 Introduction

In the context of growing recognition of the need for more environmentally-sustainable routines and practices in every type of human activity, spillover effects have often been evoked as an important contributor (Greene et al., 2024; Salciuviene et al., 2024; Yang et al., 2021). Even if many organizations and groups are working on these essential changes, each promoting change to a few behaviors in one setting at a time would be far too slow; changes need to expand beyond the original practice to other practices and settings. We may be able to implement changes in such a way as to intentionally encourage this spillover.

Spillover is generally defined as when the performance of one behavior affects the probability of engaging in (positive spillover) or disengaging from (negative spillover) a second, related behavior in the same situation, or the same or a related behavior in another context (Isbanner et al., 2021; Truelove et al., 2014). For example, a worker who was trained to reduce energy consumption on the shop floor by turning off machinery that is not in use may also turn off unused equipment or lights when participating in work meetings, and/or may be more likely to do the same at home, even if there has not been training or direction about those behaviors. Spillover has been the focus of significant research efforts in sustainability because when it occurs, investments in more sustainable routines become more effective and efficient by also increasing sustainability in other contexts. Understanding spillover better can also contribute to our knowledge of behavior change across domains (Dolan and Galizzi, 2015).

There are still significant gaps in understanding the factors that have an influence on whether, how, and when spillover occurs (Galizzi and Whitmarsh, 2019; Greene et al., 2024). Improved knowledge about these factors could be used for tailored interventions which promote spillover, contributing to more effective implementation of environmentally-responsible behaviors (ERB) across contexts and over time (Frezza et al., 2019; Henn et al., 2020). This paper reports on a first qualitative study applying a new framework to understanding spillover effects, offering insight into the mechanisms that may contribute to or impede positive spillover of ERBs, and other desired behaviors.

When research on promotion of ERBs began, it was primarily focused on directly influencing behaviors through changing socio-material aspects; through instruction and by creating regulations and/or providing equipment or objects that facilitated or required the desired change. However, these approaches can create reactance, a negative reaction to feeling one is losing the freedom to choose, which can reduce adoption of the desired attitude or behavior (Steindl et al., 2015). Individuals may feel they are performing more sustainable behaviors only to avoid a penalty or because they no longer have the option to do otherwise (Isbanner et al., 2021; Jakovcevic et al., 2014). Reactance appears to reduce positive spillover or create negative spillover. Even with gentler behavioral “nudges” intended to influence the target behavior, which create less risk of reactance, there may be little spillover (Kuhn et al., 2021).

Most current research on the promotion of more sustainable behaviors, including spillover, uses mainstream social-psychological approaches. In these the focus is on the individual, under the assumption that personal change is the key component for consistent and widespread application of sustainable behaviors (Brügger and Höchli, 2019; Henn et al., 2020; Kaiser and Lange, 2021). Both conscious and unconscious processes are likely involved in spillover, since recall and report of spillover *per se* are often inconsistent (Isbanner et al., 2021; Nash et al., 2019). Ye et al. (2022) consider that a process starting from a more-sustainable behavior may lead individuals, through both increased environmental knowledge and experiences of pleasure in connecting with other participating individuals and with nature, to the willingness to carry out more such behaviors.

Since theories of identity have frequently been used to understand the adoption (or non-adoption) of ERBs (e.g., Jaspal and Breakwell, 2014; Jugert et al., 2016; Uzzell and Räthzel, 2009), some studies have sought to explain the mechanisms of spillover via identity approaches (Thomas et al., 2016; Van der Werff et al., 2014; Whitmarsh and O’Neill, 2010). Others focus on attitudes (Brügger and Höchli, 2019; Henn et al., 2020; Kaiser and Lange, 2021). Many of these studies have measured the strength of pro-environmental identity or attitudes to show how this relates to ERB and/or spillover. Recent research has shown that the strength of the environmentally-concerned attitude or identity appears to interact with the personal “costs” of more sustainable behavior, such as time, effort, adaptation, inconvenience, social friction, and money. This combination then influences whether the individual will carry out the ERB or whether there will be positive spillover of ERBs (Brügger and Höchli, 2019; Kaiser and Lange, 2021). However, there has not yet been a more detailed explanation of how this interaction occurs, and what might lead individuals to tolerate higher “costs” and persist with more ERBs. A great deal of previous research has also been based on assessment of behavioral intentions and/or agreement with policies that support sustainability, rather than on real-life situations.

The “costs” of more sustainable behaviors (such as time, effort, adaptation, inconvenience, social friction, and money) are the individual’s perceptions of some of the socio-material factors present within the context of the behavior. Greene et al. (2024) noted the need for better consideration and more research on social-material factors as they influence the adoption of environmentally-friendly consumption patterns, such as circular economy principles. There has been some recent research specifically on these factors, such as Jaeger-Erben et al. (2021) showing that adoption of more ERB, such as repairing rather than replacing objects, is reduced when socio-material components make this more difficult, so that behavioral and financial costs are perceived as high.

Socio-material components directly impact the likelihood of spillover of ERBs as well. In a review of the literature, Rabiu and Jaeger-Erben (2022) noted that socio-material factors are central to whether circular consumption practices (such as reducing and reusing) become habitual and integrated into individuals’ lives, or remain occasional. This habitual aspect can be considered an influence on spillover. Littleford et al. (2014) showed that there was an influence of how easily a behavior could be controlled (e.g., with automatic settings) and how similar equipment and conditions were between workplace and home on whether there would be spillover of energy use reduction behaviors. Support across settings, whether organizational or social, for new, more sustainable behaviors also has been shown to be an important factor in whether spillover will occur between home and work (Rashid and Mohammad, 2011). A recent study analyzed spillover effects of sustainable consumption behavior in two contexts, home and work (Salciuviene et al., 2024). The authors note the need for programs that provide materials, information, and services (which are socio-material factors) that facilitate people’s engagement in sustainable consumption behaviors in new contexts.

The results of these studies suggest that a clearer and more detailed understanding of the integration of situational/socio-material factors with individual aspects may be useful to better explain spillover effects in general, and therefore to more effectively promote more sustainable behaviors (and other desired practices/routines) and their dissemination. Salciuviene et al. (2024) highlight that new conceptual frameworks such as the one developed by Frezza et al. (2019) that combine identity construction and socio-material factors should be applied to achieve a more detailed and complete understanding of spillover effects.

The framework developed by Frezza et al. (2019) combines Identity Process Theory (IPT) and Social Practice Theory (SPT) to help better analyze and support both changes to routines toward greater sustainability in single contexts (see Frezza and White, 2024) and spillover effects between contexts and situations. The components of the framework are explained in the following section.

Social Practice Theories (SPT), a broad family of social theories (Nicolini, 2012), have been developed over the past five decades, with contributions from different, complex and diverse perspectives. In common, they have criticism of the hyper focus on individual consciousness and agency (Reckwitz, 2017). They argue that the focus should be on social practices, not on human behavior (Reckwitz, 2002). Reckwitz (2002) defines a practice (a way of working; cleaning; consuming etc.) as “a routinized type of behavior,” made of various and interconnected elements, socially shared.

The elements that constitute practices (which are socio-material factors) are frequently divided into three groups: competencies (skills, know-how, techniques, knowledge); materials (objects such as machinery and material, technologies, infrastructure, the body itself, employee time); and meanings (ideas, emotions, aspirations, expectations) (Reckwitz, 2002; Shove et al., 2012).

Because SPT consider individuals primarily as carriers of practices, they have limitations and gaps, especially concerning the role of individuals in the maintenance, adaptation or abandonment of practices and routines. A deeper discussion of SPT overtakes the scope of this paper (please see Jaeger-Erben et al., 2021; Nicolini, 2012; Schatzki, 2015; Shove et al., 2012).

Identity Process Theory (IPT) considers identity as a product of an on-going and continuous process, while the individual acts and interacts with social, material and contextual structures and processes (Breakwell, 1986). Identity construction is regulated by two internal processes; “assimilation-accommodation” and “evaluation.” Assimilation-accommodation refers to the absorption of new elements into current identity structures (assimilation) and the adjustment of identity structures to include the new elements (accommodation). Evaluation refers to the attribution of value to identity elements (Breakwell, 2010), which can shift through this on-going process.

These processes are guided by at least four principles (continuity, distinctiveness, self-esteem, self-efficacy), which can be described as desirable states for identity, and which may involve perceptions of the self and/or of groups with which the individual identifies. For continuity, the individual seeks to achieve and maintain perceptions of self-consistency and/or group-consistency of attributes and behaviors across time and space. For distinctiveness, the individual seeks to achieve and maintain a sense of uniqueness and differentiation from other individuals and/or groups. For self-esteem, the individual seeks to achieve and maintain feelings of personal and/or group worth and for self-efficacy, the individual seeks to achieve and maintain a sense of their own/their group’s competence and control over life and situations (Breakwell, 1993).

In their daily lives, activities and choices, individuals seek to reach, maintain, and when necessary, restore satisfactory levels of these principles. New information or alterations in situations can threaten the desired level of the identity principles, leading to feelings of dissatisfaction or insecurity which are perceived as aversive, uncomfortable or distressing (Breakwell, 2021). This leads to attempts to gain or get back to satisfactory levels of the principles, which can occur through a return to former conditions, or through changes in beliefs, attitudes, behaviors, and/or physical or social settings. In this way, threats to identity principles can create barriers to desired changes (Murtagh et al., 2012), or opportunities for those changes (Frezza and White, 2024). IPT highlights that social changes affect identity construction; identity construction affects people’s actions; and, simultaneously, actions (re)shape identity (Breakwell, 1993).

**Figure 1:**
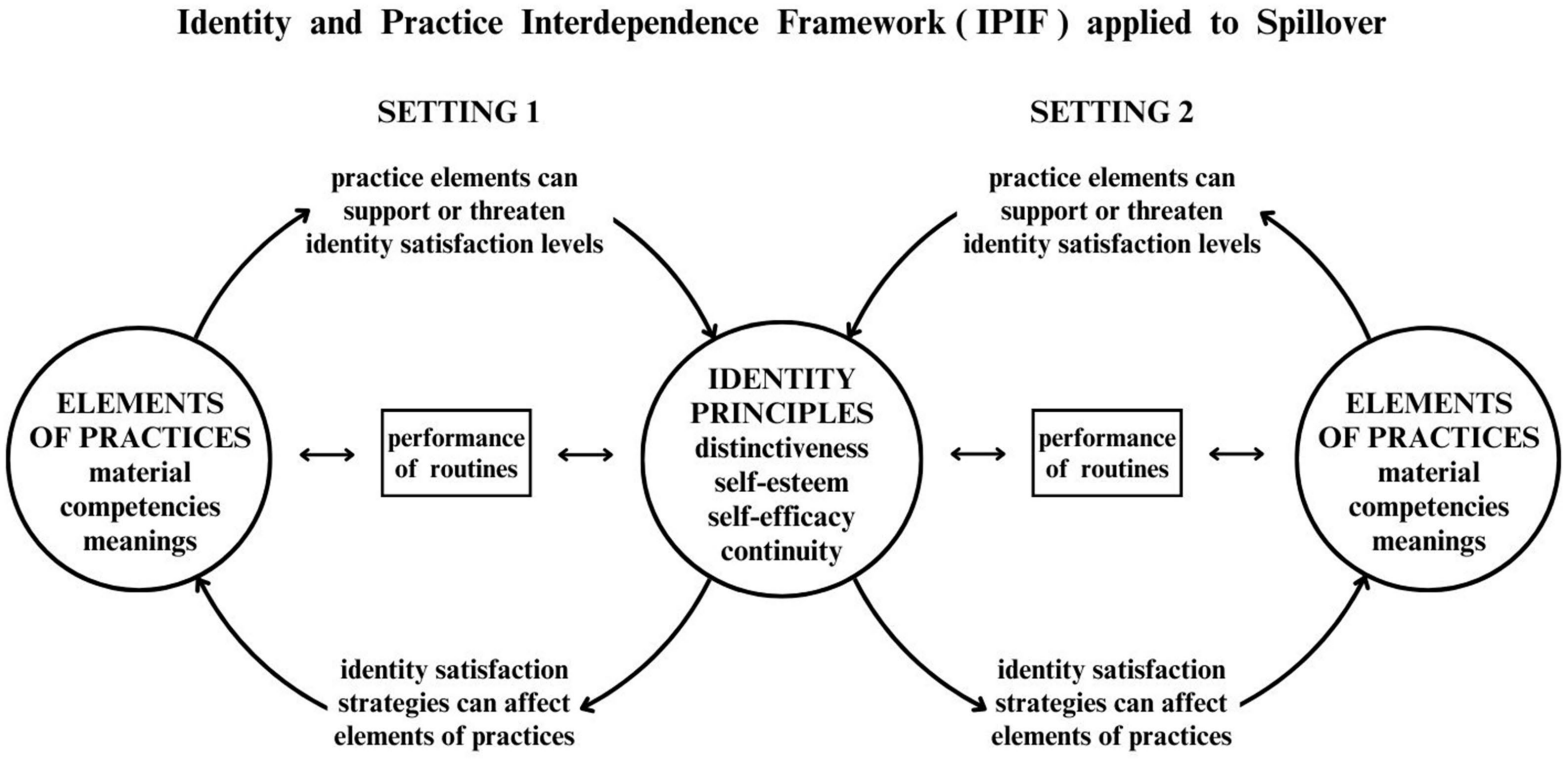
Identity and Practice Interdependence Framework (IPIF) applied to Spillover.

Frezza et al.’s (2019) framework (see Figure 1) integrates aspects of SPT (the practice elements of materials, competencies and meanings) and of IPT (the identity principles of self-esteem, self-efficacy, distinctiveness and continuity), to support analysis of the occurrence of spillover, overcoming dichotomous perspectives which focus on either the individual or the social structure.

This framework identifies aspects that can be studied empirically, taking into account the complexity of routines and changes to routines. It is important to keep in mind that the dynamics and interactions postulated by this framework will often not be conscious ones; their occurrence can be recognized in how routines are carried out, as well as in the ways people talk about themselves and their routines.

Better understanding of these processes may lead to improvements in interventions, to encourage positive spillover of more sustainable behaviors and routines. One section of the framework was recently applied to the analysis of the implementation and adoption of more sustainable routines in the workplace (Frezza and White, 2024), without looking at spillover effects.

The general research objective of the current study was to;

Investigate influences on spillover effects through the integration of principles of identity construction and elements of social practices.

The research specific aims were to;

1. Identify the occurrence of spillover of sustainable routines from the worksite to the private domain.
2. Characterize the role of the elements of practices for the spillover of sustainable routines.
3. Characterize the role of the principles of identity construction for the spillover of sustainable routines.
4. Explore how interactions between the elements of practices and the principles of identity construction help to understand whether and how spillover may occur.
5. Confirm whether the IPIF can explain influences on spillover, beyond what approaches focusing either on identity elements or socio-material factors can already provide.

## 2 Materials and methods

Because this was the first empirical study applying the IPIF to spillover, an exploratory approach was prioritized, using qualitative methods to identify and value participants’ perceptions, knowledge and experiences around this complex and dynamic topic. This approach has been recommended as a complement to quantitative research on spillover (Galizzi and Whitmarsh, 2019), especially to provide insight into the factors involved, including those that contribute to positive spillover and to barriers or resistance.

Focus groups were used for data collection in the workplace, as the group dynamics that occur can help participants explore and clarify their views, and may encourage quieter participants to speak up in exchanges with other participants. Semi-structured interviews were used in the home settings, to allow participants to express themselves as they preferred and focus on aspects they perceived as most important, while ensuring that specific topics were covered with all home participants. Onsite observations in the workplace and home settings may have improved participant recollection of ERBs and of spillover, and also allowed the researcher to see some of the material arrangements impacting adoption of ERBs.

There were two levels of data triangulation, which contributes to clarity and confidence in understanding complexity (Thurmond, 2001). The first is having four types of data gathering; focus groups at the company, onsite observations at the company, home interviews and onsite observations at participants’ homes. The second is different groups of participants involved; three types of employees in separate focus groups (management, office, and shop-floor), and two types of participants for home interviews (employees and family members).

### 2.1 Selecting the company for the field research

The research project aimed to investigate a large Brazilian company that had implemented clear and comprehensive internal socio-material measures and changes to facilitate sustainable production and consumption routines, in order to determine whether spillover to the private domain occurred, and how. To identify an appropriate research field, three Brazilian professionals with experience in management consulting in sustainability were asked for suggestions. Four major companies with a demonstrated commitment to sustainability were recommended, each operating in distinct sectors across three different regions of the country: (i) engineering and large-scale construction projects; (ii) development and manufacture of agricultural power tools; (iii) development and manufacture of technology and machinery for water and sanitation systems; and (iv) industrial steel production.

An invitation letter detailing the research project was sent to contacts at each of the recommended companies. Three companies responded: one declined, another expressed initial interest but subsequently declined, and the third, the steel-producing company, agreed to participate after a series of clarifying communications. Data was collected at one of their steel plants with a total built area of 7 million m^2^, located in south-eastern Brazil. Institutional and sustainability reports published between 2008 and 2019, publicly available on the company website, were reviewed and provided important data for the research.

It is important to note that the company where data collection was conducted did not influence any research decisions, procedures, or outcomes. The company was supportive, providing space and conditions for employee participation in focus groups conducted at company installations. The company did not provide any direct financial support for the research project or the researcher, had no access to the raw or analyzed data, and saw research results and conclusions only when they were published.

### 2.2 Data collection—phase 1

Phase 1 took place in 2017. In recruiting participants, no specific determinants, such as gender or length of employment, were set. The sole exclusion criterion was that participants had to be at least 18 years of age. Participation was entirely voluntary, with interested individuals responding to an invitation letter disseminated through the company’s internal communication system by the human resources department. The 30 employees who agreed to participate were divided in three focus groups according to their functions/positions at the company; focus group 1 had 11 participants from management and planning staff; focus group 2 had 11 participants from administrative and office staff; and focus group 3 had eight participants from shop-floor staff. This separation was intentional in order to avoid possible constraint as a result of hierarchical differences, which could affect employees’ comments. Focus groups were conducted at the company, in a private room with only the researcher and the participants, and lasted from 60 to 90 min., with audio recording. The focus group discussion guidelines can be found in Supplementary Table S1A. During this phase, onsite observations at the company were also carried out with three two-hour visits to the plant, which enriched the data. The researcher was provided guided tours of various areas within the company, including offices, employee pantries, restrooms, workshops, production sectors, water stations used for cooling the production process, employee canteens, and training rooms. In these settings, the researcher observed different situations, photographed elements deemed relevant to the study, and engaged in informal conversations with employees.

### 2.3 Data collection—phase 2

Phase 2, also carried out in 2017, consisted of semi-structured interviews and onsite observations conducted at 15 employee homes. Of the 30 participants in the focus groups, 12 accepted the invitation to participate in the home interviews. Three employees who had not participated in the focus groups also offered to participate in the home interviews. In eight of the fifteen residences, a member of the employee’s family also participated. The guidelines for home interviews can be found in Supplementary Table S1B. Photos were taken when participants showed the interviewer aspects of their home routines related to the points that they were discussing.

### 2.4 Data analysis

Considering the complexity of the data and the fact that IPIF creates initial categories, a deductive reflexive thematic analysis (TA) was developed, to identify relevant patterns and to interpret the findings (Braun and Clarke, 2012). Atlas.ti 24 was used to organize and code the data. Categories and codes for content analysis around reports of spillover were derived from the IPIF components (see Figure 1). See the table of categories and codes in Supplementary Table S2. Both data collection and analysis were conducted in Portuguese, the first language of the interviewees and the interviewer. Quotes from the data that have been included in this paper were translated by an English native speaker.

Validation of analysis was carried out through independent analysis of multiple samples of the data used for this section of the project, by another researcher experienced in qualitative research and familiar with Atlas ti. There was generally very good agreement between the two analyses, likely because analysis was based on the IPIF components.

## 3 Results

The core of the analysis was to better understand the processes of spillover, using the IPIF. In this section the occurrence of spillover is confirmed, and how practice elements were involved, how identity principles appear to have been engaged, and how these factors interact to influence the occurrence of spillover are described.

In the focus groups, participants were asked about the sustainability-related programs, initiatives and routines implemented within the company. They spoke at length about these, some of which were company initiatives while others were developed by the employees themselves. The groups were then asked to think and comment about whether there was something particularly relevant that they had “taken home” based on workplace experience, company campaigns, learned skills, concerns raised at work and so on. Waste and the use of energy and water were queried directly. The same topics were addressed with interview participants in their homes.

The results showed that there were spillover effects from the company to the private sphere of employees, concerning sustainability efforts. Participants also mentioned spillover concerning safety, health and social responsibility practices at the company. The occurrences shown in this article were chosen as particularly clear examples, however there were many more such instances mentioned by the participants. A total of 58 occurrences of spillover from 23 individual participants were identified in the data, where participants clearly stated the connection between work experiences and alterations of home routines (see Supplementary Table S3).

### 3.1 “I think we are doing our part”: adapting routines

The following participant statements about changes to routines at home were made in focus group 3, right after the discussion of a major company initiative to use water sustainably;

Matheus; *I think that here, everyone at home has a washing machine. I'm pretty sure that everyone reuses the water from the washing machine: at the end of the process, you take a bucket to store the water to wash the laundry area. I took a course [at the company] for six months, and we talked about […] reusing water, to wash [work] areas, vehicles*.

Lorenzo; *I think we are doing our part*.

**Figure 2:**
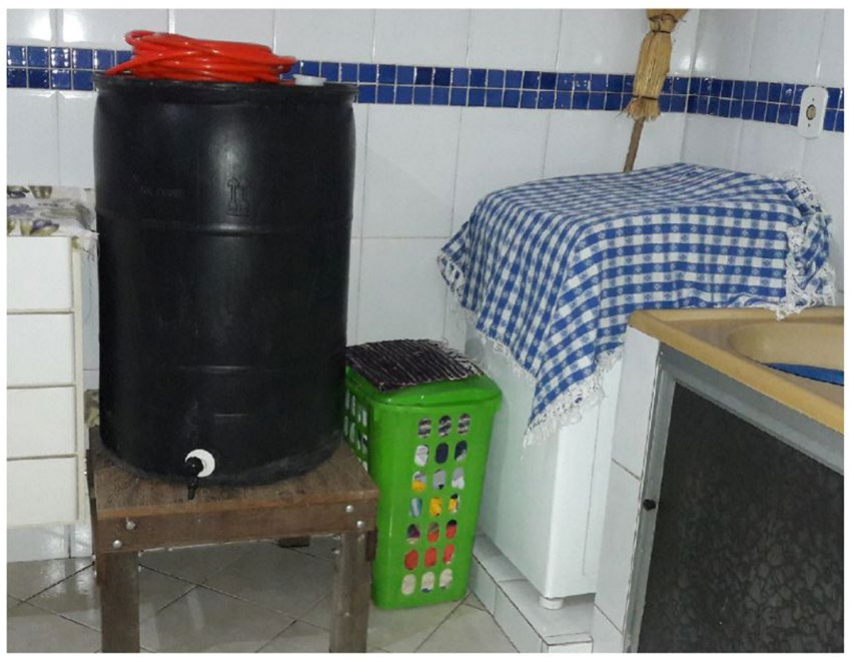
Arrangements made by participant Mônica at home; the installation of a container to collect and reuse water drained from the washing machine.

**Figure 3:**
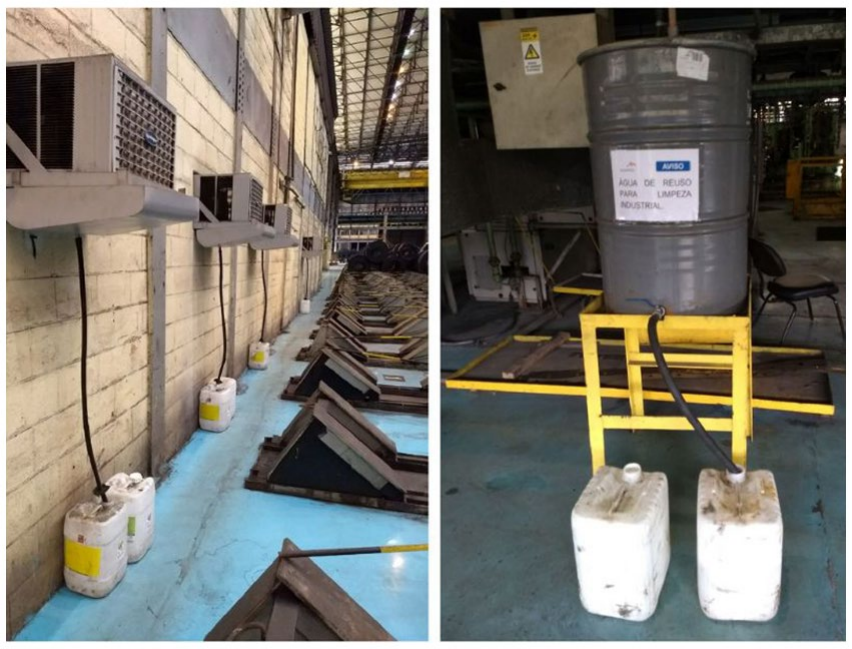
Arrangements made at the company; the installation of a system to collect the water eliminated by the air conditioning equipment, to re-use it for site cleaning.

In focus group 1, after discussion of company initiatives to use waste water for cleaning, participant Sarah proudly explained how she and her husband installed a container in their home to collect water from the washing machine, to reuse it to wash the patio. During the home interview, Mônica explained that she made similar arrangements, installing two containers in her house. Figure 2 shows the changes made by participant Mônica, and Figure 3 shows the work setting arrangements, which involve waste water from air-conditioning units.

**Figure 4:**
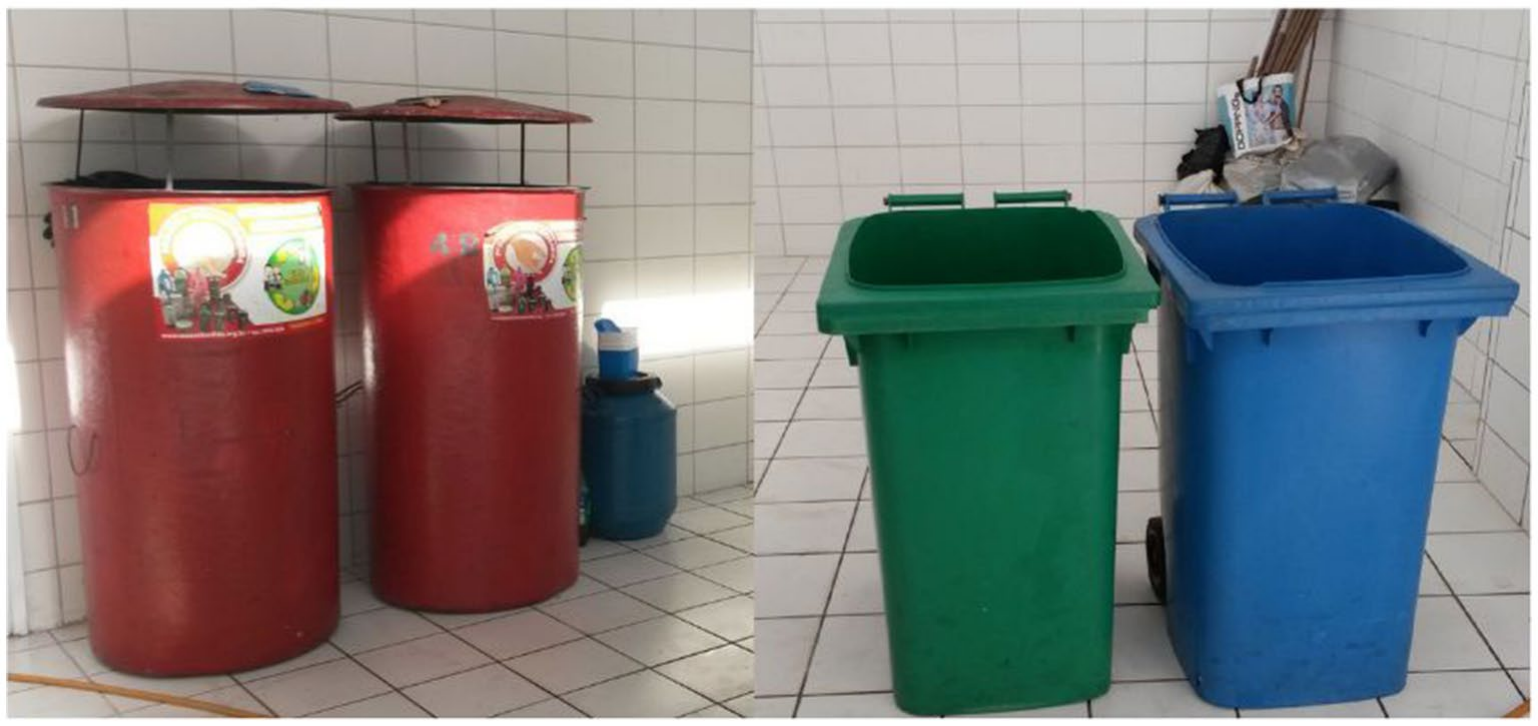
Arrangements made in the private domain to separate waste and dispose of it more sustainably. A room with bins for selective waste disposal in the condominium where participant Rafael lives; plastic bottles and aluminum cans (red containers); wet garbage (green and blue containers); and oil (blue cylinder). Behind the blue and green bins, there is an area for paper and other recyclable materials.

Another participant, Rafael, when interviewed at home, also commented on many changes he had made in the condominium he lives in; construction of a shed for waste selective disposal, with specific containers for different types of materials (see Figure 4); installation of more efficient and energy saving systems in the common areas; implementation of strategies to reduce water consumption to clean common areas and the swimming pool. He observed that everything he “brought” to his apartment and the condominium, he “took a lot from the company, from the projects […],” from what he had experienced, seen and learned there. During this onsite observation, it was possible to see the participant’s pride in these improvements, in his tone of voice, explanations, and facial expressions.

These quotes and photographs clearly show spillover. Identifying the practice elements is straightforward; material (e.g., equipment; machine; containers; system); competencies (e.g., taking courses; know-how to elaborate and to implement projects; ability to find solutions using the resources and conditions available); and meanings (e.g., reuse; reduce; doing one’s part; the workplace culture). Being able to recognize environmental problems and to plan and engage in initiatives to improve sustainability at home seems to provide ways for participants to reach satisfying levels of identity principles; self-efficacy (e.g., recognition that they are able to make changes); self-esteem (e.g., pride in applying solutions for sustainability problems); distinctiveness (e.g., identification with a particular culture; recognition that they are doing their part); continuity (e.g., observation that their actions are part of day-to-day routines; the effort to make small and big changes both at work and at home). In this example, we can also observe how routines bundle; air conditioning and cleaning; doing the laundry and cleaning.

In the workplace, participants noted reduced water consumption through the installation of clamps on all on-site water faucets. Some commented on their discomfort when unable to carry out this more sustainable routine outside the work domain, apparently because continuity needs were not satisfied;

Sarah; *It’s funny, you start to notice it in other places! For example, I go to the shopping center and I start to use the faucet [in the shopping center restroom] that doesn’t have the clamp, and there’s that ton of water coming out. It starts to bother me*.

Luís; *It’s impressive how much it becomes bothersome when people don’t do anything! It’s impressive*!

There appears to also be frustration of self-efficacy, as in this non-work setting the participants could not control the amount of water flowing. The second comment shifts the focus of the lack of spillover; this participant is bothered because others do not create conditions for the more sustainable routine. This may be a reaction to the threat to distinctiveness that arose when seeing themselves wasting so much water.

### 3.2 “I take it with me. It’s a habit!”: related routines

When discussing the impact of company initiatives on the private domain, one action in participants’ private lives that was commented on several times was the habit of taking single-use plastic bags provided (without charge) by supermarkets, to carry purchases. Participants did not connect this to any specific work routines, but related it to company initiatives to improve sustainability in general. In focus group 2, participants said this;

Alice; *I speak for myself. I use a minimal number of plastic bags nowadays at home. My bathroom bins are no longer lined with plastic bags. I collect the waste and only put it in the final trash. I go to the supermarket with those huge eco bags of mine […]. I arrive at the supermarket with a bunch of eco bags and people look at me and say “Wow! I forgot mine at home!” They see this as a good practice. They look at me and say, “Wow, how cool!” Then, they ask where I got them. But, when you go to the supermarket, you see very few people using them*.

Manuela; *Yes! Take them in the trunk of the car*.

Alice; *I have a tiny eco bag that I keep in my purse*.

Manuela; *When I go to the bakery, I take it with me. It's a habit!*

In the comments above we see spillover in routines related to waste and around reduction of the use of plastic. We can recognize the processual interactions between elements of practices and identity principles. When participant Alice explained that she had started carrying eco bags it is clear that she dealt with a set of identity feelings and had to make new configurations of practice elements (material, competencies and meanings) to adopt this new routine. Material elements also appear when Alice explained that she had stopped using the single-use plastic bags to line the garbage bins in the bathrooms of her house. This differs from the usual routines in the domestic context in Brazil, where the use of free supermarket plastic bags to line every sort of rubbish bin is very common. These small bags are then each tied up and thrown in a larger garbage bag or collection bin.

Competencies (e.g., know-how of alternatives to the plastic bags and for disposal of garbage); and meanings (e.g., resource reuse; good practice; the need to reduce plastic waste) are highlighted. Engaging in changes, participants seem to reach satisfying levels of identity principles; self-efficacy (e.g., being able to reduce plastic consumption/waste); continuity (e.g., keeping the bags in the car and in the purse to use whenever shopping); distinctiveness (e.g., very few people use eco bags); self-esteem (e.g., pride in describing personal successes in good practices). Interestingly, the issue of the plastic bags bundles different routines (shopping and waste disposal) that are impacted by this spillover.

Some narratives show that more sustainable routines can create strong reactions that encourage spillover. For instance, in one of the home interviews, the participant said;

Esther; *Everyone knows me [at the bakery]. [The attendant] doesn't put [my purchases] in plastic bags anymore. [For other] people there, they put [the bread] in a paper bag, then put it in a plastic bag. Oh, my! I can't stand it! This also happens in other stores; they wrap in tissue paper, put it in a box, then in the bag! When I buy stuff, I have a bag, so I tell the attendant; ‘There's no need to wrap it. I carry it in my bag.’ This is natural […]. Bags from the supermarket, I reuse them*.

Esther’s comments show that she is bothered by the over-packaging she witnesses, and feels she has to avoid it. Her more sustainable routine requires certain competencies (e.g., the understanding that consumption and waste are environmental problems; knowledge of ways to reduce consumption of single-use packaging); meanings (e.g., reduction of waste; reusing things); and materials (e.g., packaging; bags). Esther showed pride in being recognized by the local shops as a person who does not take the single-use bags. Her emphasis on being distinctive is clear, and she may see herself as offering an example of a different type of routine, for both store employees and other shoppers, a source of self-esteem.

At the end of the quote, Esther admitted she sometimes takes the plastic supermarket bags. In this case, it seems that the participant’s satisfaction of the identity principles may be threatened by recognizing this exception to her environmentally-responsible routine. Noting that she reuses the plastic bags may be a way to restore satisfactory levels of continuity and distinctiveness.

### 3.3 “I’d make myself do it, I’d have to”: struggling with elements of practice

In several interviews, participants mentioned they would like to dispose of waste at home in the same way it is done at the company (where there is extensive and consistent selective collection of waste). Participants explained they are unable to do the same in the private sphere, due to lack of material and space;

Manuela; *At the plant we manage it all. Organic waste from fruit, paper, lots of paper, there’s so much paper used, and glass. But there [at the company], there are five different bins, I can’t have that at home. I’d have to put it on top of my own head! There’s barely room for the garbage bin and I’ll tell you, I don’t separate wet garbage from dry. I just separate plastic bottles made of PET and aluminum cans.*

**Figure 5:**
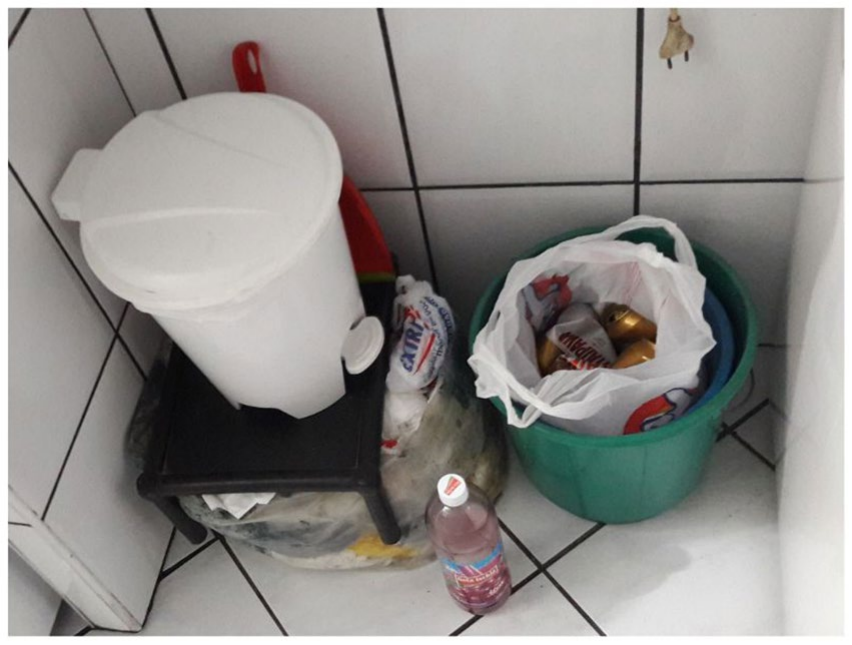
Arrangements made at home to separate waste sustainably made by participant Manuela; plastic and aluminum are kept separate from the rest that will go to landfill.

We observe the interaction between the salience of identity principles in Manuela’s quotes and elements of practices. Feelings of continuity are highlighted when she expressed her wishes to perform waste routines at home in the same way she does at work. At her home, she showed her efforts to make material arrangements to separate waste; plastic and aluminum are kept separate from the rest that will go to landfill (see Figure 5).

This same employee participated in focus group 2. There she mentioned that the company generates a lot of paper waste, which is donated to a non-governmental organization (NGO) that provides social services to underprivileged children. In her home interview, Manuela explained that she saves PET bottles and aluminum cans to donate to an organization that uses them to finance wheelchairs. With satisfaction, Manuela said that she saw wheelchairs being delivered to those who needed them. Here, we also observe the interaction of elements of practices and identity principles. By engaging in actions that involve both environmentally responsible and socially responsible meanings (donation, helping those in need) she manages to achieve positive self-esteem and a sense of self-efficacy (she sees that her actions do have positive results). The connection she pointed out between the routines she participated in at work and those at home shows the desire to achieve and maintain satisfactory levels of distinctiveness and continuity, which appears to be triggering spillover.

This participant also explained that she would find a way to separate wet garbage from paper, if she knew that the paper would be recycled;

Manuela; *I would make myself find a way to separate the paper, here in my place […] For sure. Even if I kept the wet garbage in that little bin, I’d keep the paper separate, quite separate. I’d make myself do it, I’d have to*.

This quote is especially interesting in how it highlights the discomfort and even distress occasioned by threats to continuity and distinctiveness. This participant expressed feeling an obligation to the more sustainable routine, frustrated by the lack of practice elements (material elements such as collection bins in the building, paper recycling pick-up by the city) which would permit her to participate in that routine. Her levels of self-efficacy are not threatened, however; if paper recycling were available, she is confident she would find a way, even with the very limited space she has available. It is possible that imagining herself carrying out that routine and expressing her intentions to do so reduces threats to the self-esteem that her ERB brings her.

In another interview, an employee and his partner mentioned similar difficulties. The employee observed that;

Felipe; *At the company, everything is recycled, separated, all sorted, all all selected! There's no way to do that here [at home], even if I wanted to […] there is no recycling [in residential areas]. There's no point for me to separate it here, since there is no separation where I dispose of the trash [in the building]. Let's say the condominium decides to spend money and buy bins to separate the trash. […] But when the city garbage collection truck arrives, they will take everything and put it in the truck, mixing everything, because there is no urban selective waste collection*.

The employee’s partner explained that they do sort waste, so that some can be picked out of the building’s trash by informal collectors who sell material for reuse or recycling;

Maura; *Regarding sustainability, we separate aluminum, the glass, more for the safety [of the collectors]. We put it in a separate and identified box so the collectors don't get hurt […]. When there is a lot of plastic waste, we put it all in one bag, because there are people who collect it. It's not the condominium or the city, they are [informal] collectors. […] My concern is to help them save time [while] going through the trash*.

She highlighted elements of meanings, which along with environmental responsibility included safety concerns and feelings of solidarity toward the informal waste collectors. We observe that in the work sphere participant Felipe showed that he has satisfactory levels of self-efficacy (e.g., everything is selected and recycled). But the lack of material conditions to do so at home does not stop them from seeking to achieve satisfactory levels of self-efficacy, by making extra efforts to perform partial selective waste disposal. It is possible that even partial performance of the desired routines can provide self-efficacy, when it occurs in the face of barriers and requires extra effort or ingenuity.

When asked about spillover from the company to the home, Felipe highlighted one specific behavior related to waste disposal routines;

Felipe; *There is a waste issue that I brought from the company. […] I got to know about it at the company and I brought it [home]. The company does not have trash bins by the toilets in the restrooms. So, used toilet paper is [discarded] down the toilet. At my mother's home, all used toilet paper was thrown in a trash bin. I always thought it was bad […]. When I started to work at the company, I saw that there were no bins for used toilet paper. In general, people say that the houses don't have the pipes to support it, that they will get clogged. I disagree, toilet paper won't influence that much, because it quickly [… falls apart]. Not here [in our home]! Here, all toilet paper goes down the toilet. I'm an activist for that*.

The employee’s partner added;

Maura; *At my parents' house it's the opposite. They don't accept it, […] they say it will clog [the pipes]. When I'm there, I throw everything down the toilet. But then my mother fights with me over it*.

This routine of flushing toilet paper is seen as reducing waste that has to be transported and ends up in landfill. Even though this specific routine may have a small environmental impact, we observe that the combination of practice elements with identity principles contributes to the spillover of sustainable routines/behaviors. In the two participants’ quotes, we can see the elements of practice; material (e.g., restrooms, toilet, paper, pipes, trash bin), competencies (e.g., knowing pipes do not get clogged; knowing how to dispose of waste more sustainably), and meanings (e.g., not to do something bad for the environment). We also verify the salience of the four identity principles. Self-efficacy is shown; at the company and at home, they can control how the routine is performed and see the results. Distinctiveness appears when they observed belonging to the group of people who know why and how to do this. We can note positive self-esteem in being “an activist about it” since they said this with pride. Continuity is observed when employees expressed satisfaction in acting at home as they do at the company, and in the participant’s quote showing her wish to continue performing this routine the same way at her parents’ home, even though her mother does not approve.

### 3.4 “I deserve some comfort”: resistance to changes

In the following quotes, we observe differences in how family members perform a routine at home;

Arthur; *In Brazil, the water we use to shower has the quality of drinking water […]. The production cost is high […]. I am aware of the resources […]. I know the scientific knowledge, the effort, the cost, the whole process […]. The company influences a lot, but individual consciousness is what makes the difference. To be part of a company that approaches environmental issues, and seeks so much to promote sustainability […]. But it won’t work if it doesn't make sense to you. When I soap myself [in the shower], I turn off the tap, so I don't waste water*.

These comments highlight elements of the practices involved in his showering routine; competencies (knowledge about the production process and its costs), material (potable water, resources needed for production), and meanings (the need to reduce waste of potable water). When Arthur said that “individual consciousness is what makes the difference,” it may indicate satisfactory levels of distinctiveness, since he has such consciousness. He also showed self-efficacy, in his confidence that he can reduce water consumption. The participant demonstrated that the specificity of his professional activity as well as the company’s approach affect the way he performs routines in his private life, showing spillover effects.

However, in another part of the interview, we observe that the participant does not always manage to perform this more sustainable routine;

Arthur; *I always teach this awareness to my children. Sometimes I do not achieve it; in the shower, they stay for one hour, playing. It is enjoyable, they sit down. One is eight and the other is five years old, it is not possible to demand full consciousness*.

Participant Liz is married to participant Arthur; she is also an employee of the company. She stated that she does not manage to be as careful as her husband in the shower. She explained that she keeps the tap running when soaping herself, as she feels cold when she turns off the tap. Arthur added that;

Arthur; *I tell her to turn off the tap. But there are moments that you have to respect. What can I do? I'm not going to turn it off for her. I think she is conscious, but she says she gets cold when she soaps up […]. I don't have this problem*.

Here we identify a set of resistances to the performance of sustainable water routines. In his role as a father, Arthur does not perform the same more-sustainable routines when monitoring his children’s shower as he does in his own showering routine. Children’s fun and his role as an understanding father are prioritized. These can be understood as alternate meanings, which may provide satisfaction of self-esteem needs in different ways than by behaving more sustainably. In the case of Liz, showering comfort also becomes an impediment to reducing water consumption. We also note that the negotiation attempted by Arthur with Liz to reduce water consumption is not successful.

Later in the interview, Arthur explained how his family deals with waste disposal, by separating recyclable and non-recyclable waste. Linked to this issue, he commented on his shopping routines;

Arthur; *I am aware [of waste issues], but I don't have the habit, for example, of going to the market and putting my purchases in a box. I bring it home in the supermarket plastic bags [single-use]. Why? Because life is busy, I'm tired and I […] think I deserve some comfort. It doesn't fit everything in the box, it's difficult to carry […]. But I am aware.*

He maintains his less-sustainable routine rather than changing to a more sustainable one, despite expressing awareness (meanings and competencies). It seems that the practice element of material (box/bags and time, plus the effort to remember) creates a cost; an inconvenience that reduces the likelihood he will engage in this behavior. He also defends his less-sustainable behavior. It may be that he was experiencing discomfort from threats to distinctiveness, self-esteem and continuity created by his admitting that his behavior was not always consistent with the more sustainable approach he is so familiar with from work, and that he does carry out at home in other situations. His explanations may be a way to manage that discomfort, by emphasizing other aspects that satisfy identity principles; he is a busy and hard-working person (self-esteem, distinctiveness, continuity) who deserves some comfort.

### 3.5 Identity and practice interdependence framework applied to spillover: a complete example of the dynamics

A participant quote, below, shows the spillover process embedded in the interdependence between elements of practice and identity principles, dynamically and holistically interacting (Table 1). The quote was from focus group 2, after participants had spent a great deal of time discussing work routines related to sustainability and company interventions and programs focusing on sustainability;

**Table 1:** Presents the analysis of this quote from Vinícius.

Work domain		Private domain	
Elements of practices	Identity principles	Elements of practices	Identity principles
**Materials:** Provision of systems and equipment necessary to ERB. Employee time to learn about and implement more ERB. **Competencies:** Awareness of environmental complexity; learning from other employees on a daily basis; understanding of waste issues. **Meanings:** The company focuses on safety and the environment; the environment is more than forests and animals.	**Self-efficacy:** The employee recognizes the company has the conditions to elaborate and implement daily routines that are environmentally relevant, and that as a worker there, he too can effectively do these things. **Distinctiveness:** The employee realizes the company’s approach is different from anything outside the company, and that it made him different too. **Continuity:** The employee learns about, performs and witnesses many sustainability initiatives at work. He perceives it as a consistent approach there, and himself as consistently performing more sustainable routines. **Self-esteem:** The employee expresses pride in what he learned and in how he changed because he works at this company.	**Materials:** Selective waste collection equipment at the condominium, which was not available before. **Competencies:** Ability to figure environmental issues out; know-how to implement changes at the condominium and negotiate these with fellow owners. **Meanings:** Reduction of water consumption is both more sustainable and less expensive. Separating waste allows recycling, which is very important for the environment.	**Self-efficacy:** The employee sees the changes as real and established; and expresses having been able to figure out the many issues involved, at least partly due to competencies gained at work. **Distinctiveness:** Unlike others, he wanted and worked toward more sustainable routines in the condominium. Now this condo group he belongs to is also different from others, in a similar way as at work. **Continuity:** The employee perceived a performance incongruence, with the recognition that company daily routines differ from his routines at home. The employee creates conditions to perform routines at home/the condominium similar to ways he can at the company. **Self-esteem:** The participant shows pride in contributing to many changes implemented at the condominium.

It is organized based on the IPIF, showing the interactions between the elements of practices and identity principles, within the spillover dynamics.

Vinícius; *Before [I joined the company], my notion of the environment was [limited to] vegetation, forest, sea. Like this issue of waste that we are talking about here, I had no idea. It has changed since I came here [to the company]. From the first moment I entered here, with the company approach, with its focus on safety and the environment, which raises this awareness, […] affecting every employee, […] the community. With this approach, when I went home, when I got home, I became really different. […] Today things are materializing, concerning water, waste […]. I figured this out because I work with this […]. So, I say, what I received here, in contact here, I took home, took to the condominium where I live. I'm the sub-manager of the condominium. [There,] we set targets for water consumption per apartment, and we signed an agreement to meet this target. We established waste selective collection, disposal, which before was not available in the building. We implemented many things in the condominium, including reducing costs. And why was this possible? As a consequence of what we had here, taking courses, everything I learned, I learned here, on a daily basis. And I took this to the condominium, to home*.

## 4 Discussion

### 4.1 Identifying spillover

The data gathered allowed the identification of the occurrence of positive spillover across contexts and/or situations, as described in previous studies (Isbanner et al., 2021; Mgomezulu et al., 2024; Nash et al., 2019). There were a variety of spillover examples in which participants clearly expressed connections between what they had learned, carried out and witnessed at work and their routines and initiatives in the private domain. The results were shown to be consistent due to the use of several data sources (focus groups, interviews with employees and family members, observations at work and in participants’ homes) that allowed enrichment and confirmation of the information gathered.

The examples provided are those that participants themselves identified as related to their experiences in the workplaces; it is likely that there were other situations of spillover where participants may not have been aware of the connection to more sustainable workplace routines, and therefore did not report on them during this study, as noted by Isbanner et al. (2021) and Nash et al. (2019). The format of the focus groups, where there was lengthy discussion of more sustainable workplace routines before participants were asked about efforts they had made to carry those types of routines into their homes, may also have increased participant awareness of the links.

### 4.2 Spillover: the role of elements of practices

In the data showing positive spillover, elements of practices were highlighted and noted in both domains, work and private life. This is consistent with the observation by Rabiu and Jaeger-Erben (2022) that socio-material conditions are an important influence on whether ERBs become habitual and integrated into individuals’ day-to-day lives, and with the formulation by Nash et al. (2017) and with Verfuerth et al.’s (2021) recommendation that support for context-dependent factors, such as information and easier access, could facilitate spillover.

The similarities between routines, material and competencies in the two domains might facilitate spillover. This is in accordance with Nash et al. (2017), who found that recognition of similar features in the two contexts, such as material or procedures, may trigger spillover. Margetts and Kashima (2017) found that similarity of the resources involved increased spillover, and Maki et al. (2019) showed that positive spillover was more common between similar behaviors than more different ones.

Meanings of reusing, recycling, reducing resource use, and helping those in need were constitutive of routines at the company and in the private domain, showing the importance of these elements for the spillover that occurred. They were also associated with other meanings, for instance, that the performance of ERB is valuable, important and worthwhile. Participants’ comments highlighted that these meanings (made available at the company) were constitutive of work routines and were transferred to the private domain routines. Maki et al. (2019) observed that when practitioners perceive that the original behavior and the potential other one share important goals (which can be interpreted as meanings) such as waste reduction or resource conservation, spillover is facilitated.

Participants expressed that they had gained significant sustainability-related competencies at the workplace. They clearly stated that such know-how was carried to the private sphere, becoming constitutive of the adapted routines performed at home. The importance of the acquisition of knowledge and skills for the occurrence of spillover effects was also observed by Lauren et al. (2016).

Elements of practices also played a role in difficulties, resistance and/or impediments to spillover. Lack of material contributed to unsuccessful or only partial adaptation of routines to be more sustainable. Nash et al. (2017) also observed that the lack of necessary materials, structure and/or infrastructure may limit the occurrence of spillover. Conflicting meanings at home were also observed as limitations on spillover, which is also in accordance with Nash et al.’s (2017) findings. Not having the knowledge or know-how was not highlighted as a reason for not performing more sustainably in the private domain. It is possible that when seeking to change home routines to be more sustainable, participants gravitated toward areas where they felt they already had the competencies.

### 4.3 Spillover: the role of identity principles

The data from this study suggests that when participants are able to carry out sustainable routines in the work domain (with the provided elements of practices), not performing more sustainably in the private domain may threaten the satisfaction of self-efficacy, self-esteem, distinctiveness and continuity. Those threats are experienced as uncomfortable or distressing, as identified by Breakwell (2021). Changing or adapting the configuration of routines at home to be more environmentally responsible may be seen as a way to reduce that discomfort, through restoration of satisfaction of identity principles. These processes are not usually ones that individuals are aware of, but the data allowed for recognition of these influences.

Consistent with Lauren et al. (2016), who considered self-efficacy as a possible moderator of spillover of pro-environmental behaviors, in this data attempts to meet satisfactory levels of self-efficacy played an important role. Participants appear to have experienced satisfaction of self-efficacy by adapting routines in their private lives to be more sustainable, a path they may have been able to recognize since they had experienced this type of satisfaction in the workplace. The results of the current study integrate satisfaction of self-efficacy into a more complex understanding of spillover.

In this data, participants showed some satisfaction of self-efficacy even with only partial success in adaptation of routines to be more environmentally responsible. Their frustration at not fully achieving their aims did not impede persistence in trying to improve the routines, as long as participants saw some satisfying results. It is possible that the level of challenge to be overcome contributed to this satisfaction. Lauren et al. (2019) suggest that experiences with easier sustainable behaviors (such as the participants in this study experienced in the workplace, where all the material elements were available) may increase self-efficacy, which then contributes to willingness to attempt more difficult ERB, such as participants in the current study attempted to implement at home.

Employees at times appeared to experience unsatisfactory levels of self-efficacy. The finding that it was difficult to persuade others to participate in spillover of more ERB is consistent with Nash et al. (2019). Participants appeared to seek ways of satisfying threatened identity principles by other pathways such as fulfilling other roles well. This is consistent with the findings of Maki et al. (2016) that perceptions of differences in responsibilities or roles in another context may reduce spillover. The IPIF provides an explanation of how this may occur, via alternate routes to satisfaction of identity principles.

The goal of satisfying the need for self-esteem appeared to motivate some participants to keep trying to implement more sustainable routines at home. This expectation of satisfaction of self-esteem needs may help explain the findings of Stangherlin et al. (2023), that individuals who perceived themselves as making progress toward sustainability goals often felt that they had not yet done enough, and sought ways to do more. The IPIF may be helpful to explain when this is likely to occur, and when not, through recognition of the interactions between practice elements and identity principles.

Participants did seem to feel the need to explain and justify incomplete or partial ERBs. Applying the IPIF, this can be conceptualized as a response to threats to satisfaction of identity principles. Threats can lead to a turn away from this path to satisfaction (Murtagh et al., 2012), or, when the path is still considered a viable one, to greater efforts in the more sustainable direction (Frezza and White, 2024), which can be identified as a self-efficacy issue.

Continuity was also identified in this data as an important aspect of the spillover of sustainable routines. In several examples, participants highlighted that their efforts and initiatives to behave more sustainably were part of a continuum from work to private domains, seen as something natural for them at this point, part of the (company’s) culture, or even a self-imposed obligation. This conceptualization may help understand the findings of Nash et al. (2019) that those with a higher environmental awareness were more likely to report spillover; the pre-existing environmental awareness may lead to a more noticeable or more distressing threat to continuity needs when in a context where the individual does not carry out as many/the same routines as in the first context. An individual who has lower environmental awareness may not experience the same level of discomfort due to the threat, it may not rise to a level where it is noticed and/or encourages adaptation of routines.

The cultural aspect leads to the fourth identity principle; distinctiveness. The idea of being like their neighbors, family members or others who had little interest in carrying out more sustainable routines may have threatened satisfaction of this identity principle. This could encourage adherence to more sustainable goals and initiatives outside work, to reduce that threat and restore satisfaction of identity principles.

### 4.4 Spillover effects: combining identity principles and elements of practices

Consideration of the data leads to the conclusion that specific conditions and factors within the original situation/context may have encouraged positive spillover of more sustainable routines to other situations/contexts, in this study. This can be understood through the processes shown in the IPIF (Frezza et al., 2019). The integration of socio-material conditions and identity process aspects offers an avenue for better understanding these influences than can be obtained with either focus independently.

The company took steps to establish more sustainable workplace routines through provision of practice elements. Without these, more sustainable workplace routines would have been rare, even if individual employees had tried to seek satisfaction of identity principles through this path. This is consistent with the results of Rabiu and Jaeger-Erben (2022), which showed that socio-material factors are central to whether ERBs stay infrequent or become habitual and integrated into people’s lives. This habitual aspect can support spillover.

The company made material practice elements available, such as equipment and time, that made certain ERBs feasible. The company also brought the practice elements of competencies and meanings around sustainability, through educational initiatives, campaigns, and by showing their willingness to invest (employee time, money) in ERBs. The occurrence of ERBs made possible by the practice elements thus created experiences with more sustainable routines for employees themselves, and as witnesses of others’ work routines.

When more sustainable routines are engaged by employees, the meanings appear to support a path to satisfaction of self-esteem needs (Frezza and White, 2024). Workplace steps to recognize and reward employee sustainability proposals and progress toward more sustainable routines also appear to have contributed to satisfaction of self-esteem needs. Interestingly, even being part of a group where other members were recognized appears to have contributed to achieving satisfying levels of self-esteem in participants who were not themselves recognized or rewarded (Frezza and White, 2024). These experiences, especially when repeated, can create a general recognition of more sustainable routines as a viable path to self-esteem. This may lead employees to seek that same path outside the workplace.

Through competencies provided at work, employees developed not only the knowledge and know-how needed to participate in more sustainable routines at work, but also the confidence that they can effectively do so. This provides satisfaction of self-efficacy needs (Frezza and White, 2024; Frezza et al., 2019). Repeated such experiences may create an expectation that the performance of sustainable routines is a reliable way to gain satisfaction of those needs, encouraging employees to use that same path to satisfaction of self-efficacy in the private sphere.

Participants discussed many examples of sustainability initiatives, efforts, education and adaptations of routines at work. These ranged from those that were very impactful and required large investments (construction of a sea water system for cooling equipment without damaging the environment) to quite minor (making note pads for office use out of discarded print-outs). Participants also noted that all levels of company staff were involved not only in implementing but also in suggesting sustainability initiatives, from top administration to shop-floor workers and maintenance personnel. Routines ranging from industrial processes to office and maintenance tasks, all the way to the staff cafeterias, washrooms, and cleaning practices at company installations had been adapted to be more sustainable, and these changes were made salient by company campaigns. Participants recognized that more would need to be done toward sustainability in the workplace over time, but they perceived consistency in terms of the investments, programs and initiatives at the company and of efforts by employees, including themselves. This perception likely makes more environmentally-sustainable behaviors a salient and realistic path to satisfaction of continuity needs for many employees. Satisfaction of those needs through this path at work may carry over to seeking the same satisfaction outside the workplace. Isbanner et al. (2021) noted the occurrence of a less-conscious process, where the more sustainable routines take time to become habitual and only then trigger spillover, without individuals making the connection between the original ERB and the newer one; the IPIF may also be useful for understanding influences on these types of spillover effects.

The recurrent framing of company sustainability efforts as different, advanced for the industry, and highly important at all levels of the company also offers a clear path to satisfaction of distinctiveness needs. Frequent experiences with satisfaction of distinctiveness needs at work through more sustainable routines could then lead employees to look for opportunities to create similar experiences in the private sphere, to continue to satisfy those needs.

In this way, the consistency and high salience of satisfaction of identity principles within the workplace may increase the likelihood that employees will also seek these paths to satisfaction outside of work, creating spillover. This may be experienced as the cycle noted by Ye et al. (2022), where a change to more environmentally responsible behavior leads to greater knowledge, which creates a sense of pleasure in accomplishing the ERB. That pleasure then encourages spillover. The IPIF explains further how this may be occurring, including that satisfaction of identity principles may be contributing to this sense of pleasure, and identifies components that may influence this process.

The IPIF allows us to postulate that if more sustainable routines in the workplace were occasional or partial, if provision of material elements required was inconsistent, if meanings or competencies were not reinforced by routines, or if sustainability initiatives were less central to the distinctiveness of the company, the workplace ERBs may not have created the same desire to continue to seek these satisfactions outside of work. Less consistent/distinctive sustainability efforts in the workplace might also reduce the internal pressures that several participants noted experiencing when they were only partially successful or were unsuccessful in implementing desired changes in private domains. This pressure appears to contribute to on-going efforts to behave in more environmentally-responsible ways in private life.

The IPIF provides a model that helps understand why the provision of practice elements of materials and competencies alone often leads to the limited success in behaviorally-focused efforts to encourage spillover, such as identified by Kuhn et al. (2021). Individuals who have changed behavior without engagement of identity principles and therefore without recognition (which can be unconscious) of ERBs as a path to this satisfaction may not seek to make the changes and efforts required to implement other more sustainable behaviors, or similar behaviors in other contexts.

Practice elements such as lack of materials or high costs can create barriers to spillover. It is possible that when the individual sees the ERB as attainable despite barriers, the more sustainable routine is still seen as a good path to satisfaction of identity needs, leading to persistence. If the barriers seem so high that satisfaction of identity needs is unlikely, the person may turn to other paths to satisfy them. Resistance from others can also be a barrier. Individuals may attempt to transmit meanings, competencies, and the recognition of these paths to satisfaction to other people in the second setting. This could reduce this barrier to continued satisfaction of identity principles through more sustainable routines. The IPIF may permit more comprehensive investigation of these processes.

The IPIF also suggests an explanation for the low level of spillover found in some recent studies (Carrico et al., 2018; Maki et al., 2019; Nash et al., 2019). In order to detect and investigate spillover, previous research has often focused on clearly discernible changes in sustainability-related behaviors in the first setting. This requires focus on a single behavior or a sudden change, so that changes in the second setting or context can be attributed to the influence of the change in the first. However, the current study seems to show that it is the fairly wide-spread and consistent application, in the first setting, of interventions to create more sustainable work routines that activates or strengthens the process of seeking satisfaction of identity principles in similar ways, elsewhere. This may be what leads to more frequent spillover and to persistence in pursuing spillover in the face of difficulties. It is likely that single changes toward more sustainable routines (through application of practice elements) in the first setting would not satisfy self-esteem, self-efficacy, continuity and distinctiveness needs in such a way as to trigger spillover. This may also explain why a single change in ERB can create somewhat higher intentions of carrying out more such behaviors (Haggar et al., 2023; Lauren et al., 2019) and greater support for pro-environmental policy (Jakovcevic et al., 2014; Thomas et al., 2019), but there is little actual spillover of behavior in this case. The IPIF allows understanding that when identity principles are not engaged, despite the provision of practice elements that create the original ERB, there will be little chance of spillover.

### 4.5 Practical implications

Consideration of the interactions of practice elements with identity principles through application of the IPIF can clarify some known factors that have an influence on spillover, as well as provide ideas that may help promote spillover of sustainable routines. Within organizations, providing a favorable environment which includes the three types of practice elements can be a way to foster more sustainable routines (Frezza and White, 2024; Süßbauer and Schäfer, 2018), which can contribute to experiences of satisfaction of identity principles (Frezza and White, 2024). We observe that a first setting can rarely provide material elements in a second situation or context. It is the other two practice elements, meanings and competencies, and their interactions with the identity principles, that can be “carried” by the individual into another setting or context, encouraging spillover. The person or group involved then looks for or creates or adapts the material conditions to carry out the more sustainable routines in the second setting. If that is not possible, this is a barrier to spillover.

Making those competencies and meanings noticeable and showing that they are considered important may contribute to the environmental values that Nash et al. (2019) noted as likely to encourage spillover, and to the relationship between strength of attitude and ERB (Kaiser and Lange, 2021) and spillover (Brügger and Höchli, 2019). The IPIF provides a more complex and detailed way to understand these processes, and how they can be supported.

Satisfaction of identity principles through the performance of sustainable routines within the first setting should be made salient, to encourage both more engagement in those routines in the original setting and spillover. Public recognition and private discussion (e.g., within work groups) can raise the salience of ERB as a path to satisfaction of identity principles. Making more sustainable routines widespread and frequent within the original setting reinforces meanings and competencies, as well as creating satisfaction of continuity and distinctiveness needs. It is important that even minor routines in the first setting be made more sustainable whenever possible; the sustainability impacts may be small, but the meanings that are carried and the self-efficacy and continuity this creates are important and may encourage individuals to seek that same path to satisfaction of needs both in the original setting and in other situations or contexts.

Pointing out the distinctiveness of both the organization and environmentally-engaged individuals within it may encourage people to seek satisfaction of that identity principle in other settings through spillover of ERB. Emphasizing differentiation between those who participate in more sustainable routines only because it is the default or is required and those who do so because it is important, essential and valuable can create a higher desire to maintain that same distinctiveness (and self-esteem) in other contexts.

As first indicated by Bandura (1982), self-efficacy can also be supported by offering models of peers (individuals the person perceives as similar to themselves in relevant ways) successfully carrying out the changed routines, suggesting adaptations to make routines more sustainable, or confidently navigating acquisition of new skills and changes in routines. Verbal persuasion from credible sources (those who are familiar with the capacities of the individuals/groups involved and the competencies required to participate in the more sustainable routines), can also make such a path salient. The IPIF gives a better understanding of how these well-known interventions to support self-efficacy can contribute not only to change in the original context, but also to spillover. The IPIF also shows that simply increasing self-efficacy, without provision of all the necessary practice elements, is unlikely to increase ERBs and spillover.

Normalizing managing barriers and challenges to more sustainable routines can also allow for satisfaction of self-esteem and self-efficacy needs through progress or incremental change. This may encourage spillover even in the face of difficulties with material elements or resistance from others. When changes to make routines more sustainable have not yet occurred or are incomplete, limiting satisfaction of self-esteem needs can encourage persistence, as suggested by Baumeister et al. (2003). It is the recognition of more sustainable routines as a feasible path to satisfaction of self-esteem needs that likely contributes to persistence, rather than high levels of satisfaction in the second context.

### 4.6 Strengths and limitations

One limitation of this study is that it was carried out at one site, at one point in time, limiting generalizability. Another limitation is that participants self-selected into the focus groups and home interviews. These conditions can result in participants who are more interested in and engaged with the topic of the research than the average of the population.

It is a strength that this qualitative study provided rich, detailed and contextual data, and analysis of data from multiple sources, allowing triangulation. Data collection at this large plant of a multinational steel company and at the homes of some of its employees provides a complex, real-world example of spillover of ERB.

### 4.7 Recommendations for further research

Future studies should apply the IPIF in other spillover contexts; within different settings in workplaces, from home settings to work, from educational or community settings to home and *vice-versa*, etc. It would also be particularly useful to gather data from individuals or contexts where spillover could occur, but does not, as suggested by Nash et al. (2019). Using the IPIF for study of these situations may clarify the applicability of this model, as well as providing a more detailed understanding of factors influencing spillover.

Quantification of the IPIF model/concepts would facilitate research to investigate the generalizability of the current results. Quantitative measures of the model elements would also make longitudinal studies more realistic, examining implementation of more sustainable routines in one situation or context and assessing model elements in subsequent occurrences of spillover.

Future studies should be carried out that focus more closely on barriers to spillover and on how threats to identity principles are created. Particularly interesting is what configuration of practice elements or paths to satisfaction of identity principles can influence whether threats encourage spillover of more-sustainable routines or lead to abandonment of that path for others. As suggested by Stangherlin et al. (2023), there should be more studies of what factors lead to complacency once some more sustainable routines are carried out, or to on-going efforts to expand ERB. The IPIF may be useful for these lines of research, as well as studies on when competing meanings or paths to satisfaction of identity principles will win out over application or spillover of ERB. The framework may also be useful for research into other domains of desired behavior change.

Contextual factors, such as social, economic or political conditions, may impact employee identity construction processes as well, so their impacts on these dynamic interactions in spillover should be examined.

## 5 Conclusion

The Identity and Practice Interdependence Framework allowed for a more complex and more complete analysis of situations of spillover than previous applications of practice theories or individualistic approaches alone. It permitted investigation of the integration of socio-material aspects with the role of individuals in these processes, and indicated some ways these may interact.

Meanings and competencies provided in one setting can be carried by individuals to another, but material elements are often not easily available in the second context. Individuals must try to find, create or adapt material elements necessary for implementation of more sustainable routines in the second setting. Practice elements alone, however, do not appear to explain how and when spillover occurs, or does not.

The mechanism of spillover, as conceptualized with the IPIF, appears to be that in the first setting, with all three types of practice elements fairly consistently available, individuals can achieve satisfaction of identity needs through repeated experiences (learning, performing and witnessing) with more sustainable routines. This leads to more sustainable routines being experienced as a feasible and salient path to satisfaction of identity principles for the individual. This in turn encourages them to seek that same path to satisfaction in the second setting or context. The desire to satisfy identity principles in the second setting, similarly to how they have been satisfied in the first, encourages spillover.

While the participants in this study showed awareness of connections between workplace routines and their efforts to adapt home routines, this process is not necessarily a conscious one, although individuals do at times notice discomfort when unable to carry out the more sustainable routines at home, and satisfaction when able to do so.

The low levels of satisfaction of identity principles created by single or inconsistent changes to environmentally-relevant behaviors in a first context appear to explain many situations in which spillover is desired, but does not occur as expected. Focus on this type of situation may also explain why research often has difficulty finding spillover effects.

These conclusions imply that when organizations, companies or governments implement changes, spillover can be intentionally encouraged. Spillover effects can then significantly contribute to more environmentally responsible routines, or other desired outcomes, in other settings or contexts. To accomplish this, the targeted routines in the first setting or context must be well-supported, frequent, made salient, and fairly consistent. There may be no shortcuts to encouraging spillover.

## Data Availability

The raw data supporting the conclusions of this article will be made available by the authors, without undue reservation.

## References

[ref1] Bandura A. (1982). Self-efficacy mechanism in human agency. Am. Psychol. 37, 122–147. doi: 10.1037/0003-066X.37.2.122

[ref2] Baumeister R. F. Campbell J. D. Krueger J. I. Vohs K. D. (2003). Does high self-esteem cause better performance, interpersonal success, happiness, or healthier lifestyles? Psychol. Sci. Public Interest 4, 1–44. doi: 10.1111/1529-1006.01431 26151640

[ref3] Braun V. Clarke V. (2012). “Thematic analysis” in APA handbook of research methods in psychology, vol. 2. Research designs: Quantitative, qualitative, neuropsychological, and biological. eds. Cooper H. Camic P. M. Long D. L. Panter A. T. Rindskopf D. Sher K. J. (Washington, DC: American Psychological Association), 57–71.

[ref4] Breakwell G. M. (1986). Coping with threatened identity. London: Methuen.

[ref5] Breakwell G. M. (1993). Social representations and social identity. Papers Soc. Rep. 2, 198–217.

[ref6] Breakwell G. M. (2010). Resisting representations and identity processes. Papers Soc. Rep. 19, 6–1.

[ref7] Breakwell G. M. (2021). Identity resilience: its origins in identity processes and its role in coping with threat. Contemp. Soc. Sci. 16, 573–588. doi: 10.1080/21582041.2021.1999488

[ref8] Brügger A. Höchli B. (2019). The role of attitude strength in behavioral spillover: attitude matters—but not necessarily as a moderator. Front. Psychol. 10:1018. doi: 10.3389/fpsyg.2019.01018, PMID: 31143145 PMC6520604

[ref9] Carrico A. R. Raimi K. T. Truelove H. B. Eby B. (2018). Putting your money where your mouth is: an experimental test of pro-environmental spillover from reducing meat consumption to monetary donations. Environ. Behav. 50, 723–748. doi: 10.1177/0013916517713067

[ref10] Dolan P. Galizzi M. M. (2015). Like ripples on a pond: Behavioral spillovers and their implications for research and policy. J. Econ. Psychol. 47, 1–16. doi: 10.1016/j.joep.2014.12.003

[ref11] Frezza M. White K. E. (2024). Promoting sustainable workplace routines: the identity and practice interdependence model. Sustain. For. 16:993. doi: 10.3390/su16030993

[ref12] Frezza M. Whitmarsh L. Schäfer M. Schrader U. (2019). Spillover effects of sustainable consumption: combining identity process theory and theories of practice. Sustain. Sci. Pract. Policy 15, 15–30. doi: 10.1080/15487733.2019.1567215

[ref13] Galizzi M. M. Whitmarsh L. (2019). How to measure behavioral spillovers: a methodological review and checklist. Front. Psychol. 10:342. doi: 10.3389/fpsyg.2019.00342, PMID: 31024368 PMC6460990

[ref14] Greene M. Hobson K. Jaeger-Erben M. (2024). Bringing the circular economy home–insights from socio-technical perspectives on everyday consumption. Cleaner Responsible Consumption 12:100157. doi: 10.1016/j.clrc.2023.100157

[ref15] Haggar P. Whitmarsh L. Nash N. (2023). A drop in the ocean? Fostering water-saving behavior and Spillover through information provision and feedback. Environ. Behav. 55, 520–548. doi: 10.1177/00139165231201371

[ref16] Henn L. Otto S. Kaiser F. G. (2020). Positive spillover: the result of attitude change. J. Environ. Psychol. 69:101429. doi: 10.1016/j.jenvp.2020.101429

[ref17] Isbanner S. Algie J. Reynolds N. (2021). Spillover in the context of forced behaviour change: observations from a naturalistic time-series study. J. Mark. Manag. 37, 703–731. doi: 10.1080/0267257X.2020.1865431

[ref18] Jaeger-Erben M. Frick V. Hipp T. (2021). Why do users (not) repair their devices? A study of the predictors of repair practices. J. Clean. Prod. 286:125382. doi: 10.1016/j.jclepro.2020.125382

[ref19] Jakovcevic A. Steg L. Mazzeo N. Caballero R. Franco P. Putrino N.. (2014). Charges for plastic bags: motivational and behavioral effects. J. Environ. Psychol. 40, 372–380. doi: 10.1016/j.jenvp.2014.09.004

[ref20] Jaspal R. Breakwell G. M. (2014). Identity process theory: identity, social action and social change. Cambridge, UK: Cambridge University Press.

[ref21] Jugert P. Greenaway K. H. Barth M. Büchner R. Eisentraut S. Fritsche I. (2016). Collective efficacy increases pro-environmental intentions through increasing self-efficacy. J. Environ. Psychol. 48, 12–23. doi: 10.1016/j.jenvp.2016.08.003

[ref22] Kaiser F. G. Lange F. (2021). Offsetting behavioral costs with personal attitude: identifying the psychological essence of an environmental attitude measure. J. Environ. Psychol. 75:101619. doi: 10.1016/j.jenvp.2021.101619

[ref23] Kuhn S. Ihmels M. Kutzner F. (2021). Organic defaults in online-shopping: immediate effects but no spillover to similar choices. J. Consum. Behav. 20, 271–287. doi: 10.1002/cb.1850

[ref24] Lauren N. Fielding K. S. Smith L. Louis W. R. (2016). You did, so you can and you will: self-efficacy as a mediator of spillover from easy to more difficult pro-environmental behaviour. J. Environ. Psychol. 48, 191–199. doi: 10.1016/j.jenvp.2016.10.004

[ref25] Lauren N. Smith L. D. Louis W. R. Dean A. J. (2019). Promoting spillover: how past behaviors increase environmental intentions by cueing self-perceptions. Environ. Behav. 51, 235–258. doi: 10.1177/0013916517740408

[ref26] Littleford C. Ryley T. J. Firth S. K. (2014). Context, control and the spillover of energy use behaviours between office and home settings. J. Environ. Psychol. 40, 157–166. doi: 10.1016/j.jenvp.2014.06.002

[ref27] Maki A. Burns R. J. Ha L. Rothman A. J. (2016). Paying people to protect the environment: a meta-analysis of financial incentive interventions to promote proenvironmental behaviors. J. Environ. Psychol. 47, 242–255. doi: 10.1016/j.jenvp.2016.07.006

[ref28] Maki A. Carrico A. R. Raimi K. T. Truelove H. B. Araujo B. Yeung K. L. (2019). Meta-analysis of pro-environmental behaviour spillover. Nat. Sustain. 2, 307–315. doi: 10.1038/s41893-019-0263-9

[ref29] Margetts E. A. Kashima Y. (2017). Spillover between pro-environmental behaviours: the role of resources and perceived similarity. J. Environ. Psychol. 49, 30–42. doi: 10.1016/j.jenvp.2016.07.005

[ref30] Mgomezulu W. R. Edriss A. K. Machira K. Pangapanga-Phiri I. Chitete M. Mambosasa M.. (2024). Understanding spillover effects of sustained adoption of sustainable agricultural practices on household resilience to food shocks: evidence from Malawi’s sustainable food systems program. J. Agric. Food Res. 16:101099. doi: 10.1016/j.jafr.2024.101099

[ref31] Murtagh N. Gatersleben B. Uzzell D. (2012). Self-identity threat and resistance to change: evidence from regular travel behaviour. J. Environ. Psychol. 32, 318–326. doi: 10.1016/j.jenvp.2012.05.008

[ref32] Nash N. Whitmarsh L. Capstick S. Hargreaves T. Poortinga W. Thomas G.. (2017). Climate-relevant behavioral spillover and the potential contribution of social practice theory. Wiley Interdiscip. Rev. Clim. Chang. 8:e481. doi: 10.1002/wcc.481

[ref33] Nash N. Whitmarsh L. Capstick S. Thøgersen J. Gouveia V. de Carvalho Rodrigues Araújo R.. (2019). Reflecting on behavioral spillover in context: how do behavioral motivations and awareness catalyze other environmentally responsible actions in Brazil, China, and Denmark? Front. Psychol. 10:788. doi: 10.3389/fpsyg.2019.00788, PMID: 31214064 PMC6558077

[ref34] Nicolini D. (2012). Practice theory, work, and organization: an introduction. Oxford, UK: OUP Oxford.

[ref35] Rabiu M. K. Jaeger-Erben M. (2022). Appropriation and routinisation of circular consumer practices: a review of current knowledge in the circular economy literature. Cleaner Respons. Consum. 7:100081. doi: 10.1016/j.clrc.2022.100081

[ref36] Rashid N. R. N. Mohammad N. (2011). Spillover of environmentally friendly behaviour phenomenon: the mediating effect of employee organizational identification. OIDA Int. J. Sustain. Dev. 2, 29–42.

[ref37] Reckwitz A. (2002). Toward a theory of social practices: a development in culturalist theorizing. Eur. J. Soc. Theory 5, 243–263. doi: 10.1177/13684310222225432

[ref38] Reckwitz A. (2017). “Practices and their affects” in The nexus of practices. eds. Hui A. Schatzki T. Shove E. (London: Routledge), 114–125.

[ref39] Salciuviene L. Dovalienė A. Gravelines Ž. Vilkas M. Oates C. Banytė J. (2024). Examining moral identity and engagement with sustainable consumption at home and in the workplace. EuroMed J. Bus. doi: 10.1108/EMJB-07-2023-0192

[ref40] Schatzki T. (2015). Spaces of practices and of large social phenomena. Espaces Temps 24, 1–14.

[ref41] Shove E. Pantzar M. Watson M. (2012). The dynamics of social practice: everyday life and how it changes. Thousand Oaks, CA: Sage.

[ref42] Stangherlin I. C. Thøgersen J. de Barcellos M. D. (2023). Behavioral spillover in the circular economy: the importance of consumer goals. J. Environ. Psychol. 91:102123. doi: 10.1016/j.jenvp.2023.102123

[ref43] Steindl C. Jonas E. Sittenthaler S. Traut-Mattausch E. Greenberg J. (2015). Understanding psychological reactance. Z. Psychol. 223, 205–214. doi: 10.1027/2151-2604/a000222, PMID: 27453805 PMC4675534

[ref44] Süßbauer E. Schäfer M. (2018). Greening the workplace: conceptualising workplaces as settings for enabling sustainable consumption. Int. J. Innov. Sustain. Dev. 12, 327–349. doi: 10.1504/IJISD.2018.091521

[ref45] Thomas G. O. Poortinga W. Sautkina E. (2016). The Welsh single-use carrier bag charge and behavioural spillover. J. Environ. Psychol. 47, 126–135. doi: 10.1016/j.jenvp.2016.05.008

[ref46] Thomas G. O. Sautkina E. Poortinga W. Wolstenholme E. Whitmarsh L. (2019). The English plastic bag charge changed behavior and increased support for other charges to reduce plastic waste. Front. Psychol. 10:266. doi: 10.3389/fpsyg.2019.00266, PMID: 30863332 PMC6399129

[ref47] Thurmond V. A. (2001). The point of triangulation. J. Nurs. Scholarsh. 33, 253–258. doi: 10.1111/j.1547-5069.2001.00253.x 11552552

[ref48] Truelove H. B. Carrico A. R. Weber E. U. Raimi K. T. Vandenbergh M. P. (2014). Positive and negative spillover of pro-environmental behavior: an integrative review and theoretical framework. Glob. Environ. Chang. 29, 127–138. doi: 10.1016/j.gloenvcha.2014.09.004

[ref49] Uzzell D. Räthzel N. (2009). Transforming environmental psychology. J. Environ. Psychol. 29, 340–350. doi: 10.1016/j.jenvp.2008.11.005

[ref50] Van der Werff E. Steg L. Keizer K. (2014). I am what I am, by looking past the present: the influence of biospheric values and past behavior on environmental self-identity. Environ. Behav. 46, 626–657. doi: 10.1177/0013916512475209

[ref51] Verfuerth C. Gregory-Smith D. Oates C. J. Jones C. R. Alevizou P. (2021). Reducing meat consumption at work and at home: facilitators and barriers that influence contextual spillover. J. Mark. Manag. 37, 671–702. doi: 10.1080/0267257X.2021.1888773

[ref52] Whitmarsh L. O’Neill S. (2010). Green identity, green living? The role of pro-environmental self-identity in determining consistency across diverse pro-environmental behaviours. J. Environ. Psychol. 30, 305–314. doi: 10.1016/j.jenvp.2010.01.003

[ref53] Yang S. Wei J. Cheng P. (2021). Spillover of different regulatory policies for waste sorting: potential influence on energy-saving policy acceptability. Waste Manag. 125, 112–121. doi: 10.1016/j.wasman.2021.02.008, PMID: 33684662

[ref54] Ye J. Yao Y. Li L. (2022). The more involved, the more willing to participate: an analysis of the internal mechanism of positive spillover effects of pro-environmental behaviors. J. Clean. Prod. 375:133959. doi: 10.1016/j.jclepro.2022.133959

