# Supplementary material for: Spillover of sustainable routines from work to private life: application of the Identity and Practice Interdependence Framework
Source: Front Psychol. 2024 Sep 26;15:1420701. doi: 10.3389/fpsyg.2024.1420701 (PMC11465110; doi:10.3389/fpsyg.2024.1420701)
Supplement: Supplementary file 1 [file Table_1.pdf]

Spillover of Sustainable Routines from Work to Private Life: Application of the Identity and Practice Interdependence Framework

Supplementary Material

1. Tables S1A and S1B: Tables with the guidelines for data collection: focus groups and home interviews.

Data collection guidelines	
**Table S1A: Focus group guideline**	**Table S1B: Home interview guideline**
1. The corporate sustainability initiatives/changes implemented, around the three spheres of practices; water, waste and energy<br>2. The impact these initiatives/changes had on routines at work;<br>3. How the employees felt about these initiatives/changes;<br>4. How and to what extent employees were involved in initiatives/changes toward sustainable practices at work;<br>5. How and to what extent these initiatives/changes and routines performed at work spilled over to private life routines.	1. Home routine activities that involved the three spheres of practices; water, waste and energy<br>2. Whether there were sustainability concerns;<br>3. Whether and to what extent there had been influence of work routines;<br>4. Intentions to make changes to home routines; facilitating factors; hindering factors;<br>5. Changes actually made;<br>6. How the participant felt about making changes and/or performing more sustainable behaviors in home routines.
