# Supplementary material for: Spillover of sustainable routines from work to private life: application of the Identity and Practice Interdependence Framework
Source: Front Psychol. 2024 Sep 26;15:1420701. doi: 10.3389/fpsyg.2024.1420701 (PMC11465110; doi:10.3389/fpsyg.2024.1420701)
Supplement: Supplementary file 2 [file Table_2.pdf]

Spillover of Sustainable Routines from Work to Private Life: Application of the Identity and Practice Interdependence Framework

Supplementary Material

1. **Table S2: Categories, codes and major subcodes used in the data analysis.** Based on the IPI Framework, aspects of the data collection, and some categories arising from the data. Analysis was carried out using Atlas.ti 24.

Categories	Codes	Subcodes
Source of Data	Focus Group 1, 2, 3<br>Home interview<br>Company documents	Employee<br>Family member<br>Site visit company<br>Site visit home<br>Photos
Spheres of sustainability routines	Water<br>Energy<br>Waste	Use/consumption<br>Conservation/reduction<br>Disposal
Routines with sustainability impacts - location	Work<br>Private life/Home<br>Elsewhere	Sector of workplace<br>Sector of private life
Sustainability routines – whose initiative	Company<br>Employee<br>Family member<br>Other	
Spillover	From work to private life<br>From private life to work<br>From elsewhere to private life<br>From elsewhere to work	Desired<br>Successful<br>Partly successful<br>Desired but unsuccessful<br>Not desired/resisted
Practice Elements (PE)	Materials<br>Competencies<br>Meanings	Existing/new<br>Present/absent/partial
Identity Principles (IP)	Self-efficacy<br>Self-esteem<br>Distinctiveness<br>Continuity	Seeking satisfaction<br>Experiencing satisfaction<br>Experiencing dissatisfaction
Individual engagement with sustainable routines	Engagement<br>Resistance	Related to IP<br>Related to PE
Other routines impacting sustainable routines	Practice elements<br>Identity principles	Facilitating<br>Hindering
Interactions between PE and IP around sustainable routines	From PE to IP<br>From IP to PE<br>Other	Facilitating spillover<br>Hindering spillover

Spillover not related to sustainability	Health<br>Safety<br>Social engagement	
