# Supplementary material for: Spillover of sustainable routines from work to private life: application of the Identity and Practice Interdependence Framework
Source: Front Psychol. 2024 Sep 26;15:1420701. doi: 10.3389/fpsyg.2024.1420701 (PMC11465110; doi:10.3389/fpsyg.2024.1420701)
Supplement: Supplementary file 3 [file Table_3.pdf]

Spillover of Sustainable Routines from Work to Private Life: Application of the Identity and Practice Interdependence Framework

Supplementary Material

1. Table S3: Table with clear incidents of spillover from work to private domain

This table presents quotes collected from home interviews (HI) and focus groups (FG) that show incidents of spillover from work to the private domain. A total of 58 occurrences of spillover from 23 individual participants were identified, where participants clearly stated the connection between work life and alterations of home routines. Sections in white are examples of spillover of routines that are very similar at work and at home. Sections in gray are examples of spillover of routines that are different from one setting to the other.

1.	**Arthur**<br><br>**(HI)**<br><br>**Different Routine**	Arthur: In Brazil, the water we use to shower has the quality of drinking water [...]. The production cost is high [...]. I am aware of the resources. I specialized in environmental engineering [...]. I know the scientific knowledge, the effort, the cost, the whole process [...]. The company influences a lot, but [...] individual consciousness is what makes the difference. To be part of a company that approaches environmental issues, and seeks so much to promote sustainability [...]. But it won't work if it doesn't make sense to you. When I soap myself [in the shower], I turn off the tap, so I don't waste water. [...].<br><br>Interviewer: [In the shower], do you [... turn off the tap] why? What is your concern?<br><br>Arthur: Because I'm not under the shower and I close it so I don't waste that water. That water is drinkable. [...] it is expensive water in every sense, [...]
2.	**Arthur**<br><br>**(HI)**<br><br>**Similar Routine**	Arthur: I'm very disciplined and I really discipline the people I'm with, no throwing [trash] in the street. For me it's the worst of all worlds.<br><br>Interviewer: And in this sense, do you think you are different from other people?<br><br>Arthur: I do, because [in the city] there are people who just trash it up, and I'm different from them. [On beaches,] people are capable of drinking beer and leaving the can on the sand, a lack of respect. [...] This lack of a sense of education regarding waste is quite annoying [...].<br><br>Arthur: Back in my childhood, we didn't have this awareness, I remember throwing things [trash] in the street, in the 80s.<br><br>Interviewer: And what favored this change?<br><br>Arthur: I think the work, the [company] campaigns, the culture you acquire.

**3.**	**Arthur and Liz**<br><br>**(HI)**<br><br>**Different Routine**	Arthur: [During shower] do you turn off the tap to soap?<br><br>Liz: Not to soap. I turn it off to apply shampoo and cream, not to lather.<br><br>Liz: In fact, I soap up under the [running] water, while rinsing my body.<br><br>Arthur: This is my achievement!<br><br>Liz: He's much more controlled, much more.<br><br>Liz: I confess that I feel cold, so I soap up with the running water. I don't get out of the shower to soap up, soaping is done under the shower, so.
**4.**	**Manuela**<br><br>**(HI)**<br><br>**Similar Routine**	Interviewer: And did you try to find out where to buy the tap [with time/volume regulator]?<br><br>Manuela: No. I'm waiting for my new property [to be ready]. I check with the firefighters there [at the company] if they do this type of external service or if they recommend someone. I like [to consult them], they know [what it's like] there. Because I think it's interesting for the sink [...]. I like the way it works [in the company].
**5.**	**Manuela**<br><br>**(HI)**<br><br>**Similar Routine**	Interviewer: And is there anything else you are thinking about installing in this new apartment?<br><br>Manuela: [At the company,] they also talk about the toilet flush time being shorter, I don't know if it's the type of valve, or if it's the time, the amount of water that is used... Then you also have this timer, which they talk about.<br><br>Interviewer: And you also saw this at Arcelor.<br><br>Manuela: Yes, in our area they have them.
**6.**	**Manuela**<br><br>**(HI)**<br><br>**Similar Routine**	Manuela: At the plant we manage it all. Organic waste from fruit, paper, lots of paper, there’s so much paper used, and glass. But there [at the company], there are five different bins, I can’t have that at home. I’d have to put it on top of my own head! There’s barely room for the garbage bin and I’ll tell you, I don’t separate wet garbage from dry. I just separate plastic bottles made of PET and aluminum cans. Other than that, there’s no way, there’s no space for that kind of waste sorting. [...] The apartments are too small for that, unless there were proper collection bins in the buildings, but there aren't.<br><br>Manuela: I would make myself find a way to separate the paper, here in my place […] For sure. Even if I kept the wet garbage in that little bin, I’d keep the paper separate, quite separate. I’d make myself do it, I’d have to!<br><br>Manuela: Look at this here, I separate it and then I make a donation and every, I think every sixty PET bottles leads to a wheelchair.<br><br>Interviewer: That little ring in the can?

		Manuela: Yes, from the can. [...] I collected it with two friends and we saw the chair being delivered, for an NGO.
**7.**	**Helena** **(HI)** **Similar Routine**	Helena: In this water crisis, the company did a very intense job. Wow! There was some informational material [distributed to employees at the company] that I distributed it. I brought it here to the building, put it in the lobby. [...] This material was really cool [...]. So, whenever I have the opportunity, I bring something, share it with people, help extend this awareness work that they do with us.
**8.**	**Alice** **(HI)** **Similar Routine**	Alice: I turn off the light, usually I do. Or, if I'm going back soon [to the room] I don't turn it off, [...] I don't think it saves that much if you turn it off and turn it on right away. [...] I think it costs more, because depending on the type of bulb, and here at home most of them are not economical bulbs, so if you turn it off and turn it on again, you end up using more [energy]. <br>Interviewer: How did you find out about this? <br>Alice: There is this issue of having participated a lot in environmental activities in the company; the courses I took, the company provided me with many courses... because I worked and implemented the environmental education program in the company. […] Yes, I've participated in many seminars.
**9.**	**Alice** **(HI)** **Different Routine**	Interviewer: Which do you think came first; the commitment within the company or this more personal issue? <br>Alice: I don't really separate the environmental from the social. But when it comes to environmental issues, my commitment to the company came first. Why? Because when I joined the company, I joined as a secretary. When I went to the environmental area, I implemented the 'Company and Community' program. And soon after our manager came, he pushed us a lot. [...] He gave lectures on environmental issues [...]. I had already trained as an educator, but I didn't know much about environmental education, [...] I was pushed by the company, motivated by the company. <br>Alice: When I worked with environmental education, [...] I planned at home to use the washing machine as little as possible, [...] wash the clothes only twice a week, [plan] the amount of clothes I put in the machine.
**10.**	**Alice** **(HI)** **Different Routine**	Alice: A lot! Very! The issue of security is one of the things that we bring home the most. I think it's something, let's say, natural! I think so, environmental issues too, but the natural thing is safety! Because my husband works shifts [at the same company], so I think he incorporates this and brings it into his daily life. <br>Alice: [...my husband] he always brings this! He says; Look, in the company it's like this, like this, we have to [do it the safe way...]; Don't use it like that, don't use that knife facing up, don't use it... understand? These everyday things, electrical and other equipment, and other things around the house.

**11.**	**Alice**<br>**(HI)**<br>**Similar Routine**	Interviewer: Compared with other people, families, friends, do you notice any difference [... in relation] to safety?<br><br>Alice: I see... I see... I have friends who are also very careful, [...]. But there are others that I see that [... are not]. Maybe, because we work in a company that [...], because [...] at the company, we act that way. For example, the use of seat belts, we already used them [there] a long time ago, before it was mandatory for everyone.
**12.**	**Alice**<br>**(HI)**<br>**Similar Routine**	Alice: The company already had this concern [on the company's internal streets]: speed, stopping at the crosswalk, waiting for people to pass [cross the street]. This culture inside was much earlier than outside. So, I always stopped, I always respected the [pedestrian] lane.<br><br>Interviewer: And how did you feel or do you feel [taking these precautions]?<br><br>Alice: Oh, I feel good. I think... this way, we are doing what is right! We are doing what should be done, [what should be] normal. We feel good about having done it because we know it is right.
**13.**	**Cecília**<br>**(HI)**<br>**Similar Routine**	Cecília: [At the company,] there are billboards, [...] screensavers, [...] the newsletter that we receive. [...] And the long-standing 'zero food waste' campaign. [...]. This is an item that I try to police myself a lot; because when we live alone [...] you end up, [... want to have] a variety of foods, which allow you to have adequate nutrition. But, at the same time, [you should buy] a small quantity so it doesn't spoil. And then, I try [...] not to waste food and there [at the company] it is something that I think is reinforced a lot. Eh, I still can't manage to not waste anything at all, but I'm also not that person who wastes a lot, no.
**14.**	**Gabriel**<br>**(HI)**<br>**Similar Routine**	Gabriel: We leave the room, we [I and my roommate] turn the light off. When we leave the apartment, we don't leave it on. [...].<br><br>Gabriel: He [my roommate] also worked at the company I work for, which cultivates a lot of these [practices], so he ended up getting that habit.
**15.**	**Gabriel**<br>**(HI)**<br>**Similar Routine**	Gabriel: [We are] doing this here [at home], the gradual replacement with LED bulbs. [...] They are doing this at the company, in all areas, they are substituting with LED.

**16.**	**Gabriel**<br><br>**(HI)**<br><br>**Similar Routine**	Interviewer: How do you see waste selective collection at the company?<br><br>Gabriel: When you leave the cafeteria, ... sometimes they give you a popsicle for dessert. When you discard the stick, the plastic, you separate it. Everyone does it, you see everyone doing it. You rarely get a bin [with] everything together. This is a clear example that we see. And here outside [the company], we end up doing that too. [...] I try to walk to a trash can and throw it in the trash. There are people who throw trash on the sidewalk. I don't do it.
**17.**	**Lorenzo**<br><br>**(HI)**<br><br>**Different Routine**	Lorenzo: And my father [who also works at the company...] changed the air conditioning [... for] a more economical one [...]. At the time, he spoke with a colleague of ours, [...] who works in the refrigeration area [of the company]. He said to buy a 220v device, [...] which is more powerful, [...] and does the job faster.
**18.**	**Lorenzo**<br><br>**(HI)**<br><br>**Similar Routine**	Interviewer: Do you see any connection between the way they use energy there [at the company] and at home?<br><br>Lorenzo: Yes. An example, when there was the water crisis here in the state, in addition to the campaign about water [at the company], this generated another campaign about electricity. Because the water crisis affected both sides. So, [at home...,] sometimes I would leave the computer screen on, [...] and my notebook would stay on almost the whole day. After that [the water crisis and the campaigns], I stopped for a while. I also had the habit of whenever I was at home, [...] the TV in my room was always on [even when] I came here [...]. to the living room [...] and the two TVs were on. So, certain things we have been working on in terms of these issues.
**19.**	**Lorenzo**<br><br>**(HI)**<br><br>**Similar Routine**	Lorenzo: My sister and I wash a lot of dishes. [...] Always, for the sake of saving water, we wet everything, pretty much, turn off the tap, soap everything and then rinse everything, really to save as much water as possible.<br><br>Interviewer: And have you always done it like this, since you were little?<br><br>Lorenzo: In fact, my father has always worked there all his life and the company has always valued this culture. So, it ends up that you repeat that a little. [...] I find it interesting that the company has always made a point [...] of what it passes on to its employees, it passes on to the family. [...] I remember that [...] my father would go to the company and show us a little bit because he had several lectures and everything. This meant a lot to a group of young people, so from when I was little, around eight or nine years old, we turned and moved one or the other and helped wash the dishes. Then we started to really create a routine [...]. So, my father and mother told us, "you're not going to waste water for nothing, don't leave the tap on and wash dishes. Turn it on, wet everything, and turn it off; use soap, and then rinse everything".

**20.**	**Lorenzo**<br><br>**(HI)**<br><br>**Similar Routine**	Lorenzo: A colleague and I traveled two years ago to spend a carnival in Minas Gerais, my sister went with us. [...] We were like this... because we didn't see a single trash bin in the city.<br><br>Interviewer: What do you mean by 'like this'?<br><br>Lorenzo: We were frustrated, because within the company there is a certain concern with waste. And it ends up that you take it into your everyday life. So, sometimes, I see people throw a piece of paper on the ground [outside the company]. And [I think;] 'put it in your pocket until you find a trash bin'.
**21.**	**Lorenzo**<br><br>**(HI)**<br><br>**Different Routine**	Lorenzo: For the company, safety comes first.<br><br>Interviewer: Did this affect you in your day-to-day life outside the company?<br><br>Lorenzo: Yes. When I joined the company, in the operations area, really at the beginning, we were more careless. And throughout the training and everything else, we see [...].<br><br>Lorenzo: An example, sometimes, to change a light bulb, I would get on a chair. Not anymore! We bought a ladder [at home] because how many times have I fallen off my chair? Afterwards, you start to create values […].<br><br>Interviewer: After falling or after joining the company?<br><br>Lorenzo: After joining the company. I'd fallen so many times before and, but... Then, you see that there are certain risks, which are not worth it. So, the company values this very much and it ends up, they ask us to take this into our daily lives.
**22.**	**Lorenzo**<br><br>**(HI)**<br><br>**Different Routine**	Lorenzo: [...] how to carry a knife, carry glasses, ok?! when dealing with electricity, another task, there is equipment made for this, a rubber glove...
**23.**	**Lorenzo**<br><br>**(HI)**<br><br>**Different Routine**	Lorenzo: [...] we had a steel mesh glove, but it tore in the middle. [...] It was for cutting meat. [...]. It prevents you from cutting yourself.
**24.**	**Murilo**<br><br>**(HI)**<br><br>**Similar Routine**	Murilo: And the paper, right? This is a culture that I brought from the company, because [at home] we used to print a lot on only one side, [...] and in color. Today, our printer at home has the same settings as the one at the company; configured black and white, front and back. [...].

**25.**	**Murilo and Júlia**<br><br>**(HI)**<br><br>**Similar Routine**	Júlia: The waste issue is such a silly thing [...]. I remember we had already walked a long distance holding a popsicle paper because we couldn't find a trash bin [...]<br><br>Murilo: On the beach.<br><br>Júlia: I also think this type of education came as an adult after Murilo experienced this [in the company].<br><br>Murilo: The thing about being careful with waste was really when I started… , it was the influence from my work at the company, [...] it came from there [the company] to here [private life…]. I took courses and everything. [... in the company] so many millions were invested in education, awareness, [...] and installation of bins.
**26.**	**Rose**<br><br>**(HI)**<br><br>**Different Routine**	Interviewer: And can you give me an example of this know-how that the company offers]?<br><br>Rose: Courses, training, [...] our screensaver this week is talking about the drought in Brazil, there is always the concern, to promote awareness about waste issues. [...].<br><br>Rose: I found out about it there [at the company], there are several engineers. I started talking to people, "Guys, I'm spending a lot on my electricity bill, I'd like to know what I can do. My electrical wiring is new. An electrical engineer has already come to the condominium, the wiring is new. How can I reduce it? " Then, a colleague of mine who is an electrical engineer came here and [guided me].
**27.**	**Felipe**<br><br>**(HI)**<br><br>**Similar Routine**	Felipe: At the company, everything is recycled, separated, all selected, all all selected! There's no way to do that here [at home], even if I wanted to [...] there is no recycling [in residential areas]. There's no point for me to separate it here, since there is no separation where I dispose of the trash [in the building]. Let's say the condominium decides to spend money and buy bins to separate the trash. [...] But when the city garbage collection truck arrives, they will take everything and put it in the truck, mixing everything, because there is no urban selective waste collection.
**28.**	**Maura**<br><br>**(HI)**<br><br>**Similar Routine**	Maura: Regarding sustainability, we separate aluminum, the glass, more for the safety [of the informal collectors]. We put it in a separate, labeled box so the collectors don't get hurt [...]. When there is a lot of plastic waste, we put it all in one bag, because there are people who collect it. It's not the condominium or the city, they are [informal] collectors. [...] My concern is to help them save time [while] going through the trash.
**29.**	**Felipe and Maura**<br><br>**(HI)**<br><br>**Similar Routine**	Felipe: There is a waste issue that I brought from the company. [...] I got to know about it at the company and I brought it [home]. The company does not have trash bins in the restrooms. So, used toilet paper is put in the toilet. [...] At my mother's home, all used toilet paper was thrown in a trash bin. I always thought it was bad [...]. When I started to work at the company, I saw that there were no bins for used toilet paper. In general, people say that] the houses don't have the pipes to support it, that they will get clogged. I disagree, toilet paper won't affect it that much, because it quickly [... falls apart]. Not here [in our home]! Here, all toilet paper goes down the toilet. I'm an activist for that.

		Maura: At my parents' house it's the opposite. They don't accept it, [...] they say it will clog [the pipes]. When I'm there, I throw everything down the toilet. But then my mother fights with me over it.
**30.**	**Felipe and Maura**<br><br>**(HI)**<br><br>**Similar Routine**	Felipe: In the company, safety is a value, safety comes first [...]. If it affects your safety, you can stop production. [...] This really has an impact.<br><br>Felipe: You pay more attention; when you see someone doing something wrong [inside and outside the company] that could go wrong [with the risk of an accident]; like someone cleaning a window, hanging off the window frame... These things, for those who don't have this culture, are a normal thing. But then you...<br><br>Maura: You will alert them. Trying to warn them doesn't mean that the person will listen, but we do our part.
**31.**	**Rafael**<br><br>**(HI)**<br><br>**Similar Routine**	Rafael: Everything I brought here [my apartment and condominium] I took a lot from the company, from the projects [...]. Regarding energy, when I moved here, the first thing I did was put LEDs in everything. [...], the entire condominium. For example, in the corridors [...] there were five light fixtures [...]. We did a lighting study, [... to evaluate] what was satisfactory. Afterwards, we removed them, I removed two light fixtures from this corridor and I installed LEDs in the other three. A slightly higher power, to bring lighting very close to what existed before. With that, I was able to deactivate two light fixtures per floor, and since there are four floors...<br><br>Interviewer: How do you connect this with your routine at the company?<br><br>Rafael: [... At the company,] we had a serious problem with light bulbs too, they burned out too frequently. I had to find people to [change the light bulbs], [...] and there was a shortage of staff. [...] We went to see with the supplier how to improve this environment [... and changed] the bulbs to LED, which last longer and save money.
**32.**	**Rafael**<br><br>**(HI)**<br><br>**Similar Routine**	Rafael: I brought many things here that I saw there [at the company].<br><br>Rafael: [About waste separation], in the apartment we actually started doing it after I had implemented it in the condominium, I brought that idea from the company. [...We made a partnership with] an NGO project. They take everything that is waste from the condominium, plastic, oil...<br><br>Rafael: Selective waste collection at the company, [...] I don't know what year it started, [...] there is separation of organics, plastic, paper, metal, glass. So, [what I implement here] is an extension of what I learned there.
**33.**	**Rafael**<br><br>**(HI)**<br><br>**Different Routine**	Rafael: For the condominium [...], I have two projects; build a twenty-five-thousand-liter tank to store and reuse waste water from air-conditioning units, rainwater [...] and pool backwash water. At the first residents' meeting, they did not approve it. But I will submit it again.<br><br>Rafael: Currently, in the condominium, I placed a valve in the pool's water intake system, so that water from the pool does not overflow. [...] Before, this water was wasted, it automatically went to the street, to the sewer. [...] And every Monday there is

		pool maintenance, so the cleaning staff uses that pool water to wash all the pool chairs, the entire area around the pool.
**34.**	**Vicente**<br>**(HI)**<br>**Different Routine**	Interviewer: How did these environmental-related trainings impact [your day-to-day life]?<br><br>Vicente: They help us, [...] we see the amount of water, how it is ... very scarce, how it's worsened by … wasting water, not reusing it. So, we bring it home ... we keep an eye on it when we take a shower, ... turn off the tap to soap ourselves, avoid wasting water.
**35.**	**Vicente**<br>**(HI)**<br>**Different Routine**	Vicente: To tell the truth, when I started working there, at the company. [...] I lived with my parents and ... we didn't ... do this differentiated selective waste collection. We put everything altogether in the same place. When I started working at the company, I came to live with my wife, [we started] this selective collection part.
**36.**	**Esther**<br>**(HI)**<br>**Different Routine**	Esther: Wow! I started to do it a long time ago! When no one talked about oil collection, I already separated my [cooking] oil. I always learned in environmental education [at the company] that oil thrown into the sewage system clogs everything, and pollutes millions of liters of water [...]. So, we informed the employees about it. How am I going to give a course to employees, provide training [...] and I'm not going to do it at home?! We have to be coherent.<br><br>Esther: I was the one who implemented it [collection of used cooking oil], it took me two years to implement it here [in the building]. People hadn't even thought about it. The collection container is downstairs [in the parking area]. When I go there, there is always a lot of oil [...]. I think that's cool, it's a seed that we're planting ….
**37.**	**Esther**<br>**(HI)**<br>**Similar Routine**	Esther: [...] society is very... they don't separate properly. There is still no selective waste disposal in my building. But when the informal collector comes by, when he goes to pick up the [recyclable material from] the building's trash, he takes my bag, which is all separated properly.<br><br>Interviewer: And has it always been like this, have you always separated [the trash]?<br><br>Esther: No. I started doing this after I started working at the company and understanding a little about environmental education. I never..., I hadn't done that before. I joined the company when I was twenty-six years old. Everything I learned in the area of environmental education was there.
**38.**	**Esther**<br>**(HI)**<br>**Different Routine**	Esther: Everyone knows me [at the bakery]. [The attendant] doesn't put [my purchases] in plastic bags anymore. [For other] people there, they put [the bread] in a paper bag, then put it in a plastic bag. Oh My! I can't stand it! This also happens in other stores; they wrap in tissue paper, put it in a box, then in the bag! When I buy stuff, I have a bag, so I tell the attendant; 'There's no need to wrap it. I carry it in my bag.' This is natural [...]. Bags from the supermarket, I reuse them.

39.	**Esther**<br>**(HI)**<br>**Similar Routine**	Esther: I even "contaminated" some people in my family. My son took a while, he was very annoyed, even today, about saving energy. He still complains with me because at night I turn off the lights in his house [...]; "Mom, you have to leave the light on because of Mateus [my grandson], ... Mom, you're annoying." I said; "there's no need." [...]. And I mentioned it because he had complained about the energy [bill]. I had a very serious conversation with him there. [...]<br><br>Esther: The company helped me with that, for sure. Before, in the offices, [...] it was not possible to turn off the lights separately. The company changed that. Today, each light has an individual switch. But it wasn't like that before, the light switch was general for the entire room. [...] It was also a way for the company to encourage every employee to turn off their light.
40.	**Sarah**<br>**(FG-1)**<br>**Different Routine**	Sarah: I think that these actions that we do here or that we know about here, within the company, we, in a way, take home [...]. Many domestic initiatives on water consumption have emerged from within the company; time bathing, we start to control that more; turn off the shower; reuse water. For example, I bought a big container to reuse water from the washing machine, to wash the terrace, clean up the dog's pee and so on.
41.	**Samuel**<br>**(FG-1)**<br>**Similar Routine**	Interviewer: And what does that mean for you?<br><br>Samuel: For me, this has become a value, [...] it is not an external obligation, it is a necessity. So much so that everything we do here, I have already taken home. I moved [residence] in February. Before I moved, I took several actions [...]; all LED lighting, which heats up less and consumes less electricity; [...] motion detectors, if there is no one around, the light turns off by itself, there is no need to [turn it off], [...] more glass to have natural light in the house. Of course, this has a cost, I could have done more. [...]
42.	**Samuel**<br>**(FG-1)**<br>**Different Routine**	Samuel: [...] reusing rainwater, we have two thousand liters of water storage capacity [at home] with just rainwater.
43.	**Arthur**<br>**(FG-2)**<br>**Similar Routine**	Arthur: And this influences [routines] at home, for example, in my apartment building, I tried to implement selective waste collection. [...] Today, in the entire building we separate dry and wet waste. We can't do paper, plastic, ..., as we do in the company [...]. Sometimes, it's the municipal garbage pickup that does it wrong, when it collects the garbage, they mix everything up.
44.	**Olívia and Murilo**<br>**(FG-2)**	Olívia: The program is in the food area; we have a goal of zero food leftovers on trays [at company cafeterias]. I see that this program has a series of aspects: one of them is actually the reduction of organic waste; the other is to create the habit of the employees really only putting on the tray what they are going to eat [...]

	**Similar Routine**	Murilo: Certainly, we have incorporated this habit at home, and it is noticeable when out our lunch, especially if we eat near other people who do not have this habit, we notice how they see it differently. Their relationship with food is totally different.
**45.**	**Yasmin** **(FG-2)** **Similar Routine**	Yasmin: Even in relation to seasoning [in food preparation], the food [in the company cafeterias] uses much less salt. And so, we end up getting used to that less salty flavor. At home, I can't use as much seasoning anymore. I like spicy food, I do! But day-to-day life [at the Company] makes us get used to it and we end up changing at home too.
**46.**	**Manuela** **(FG-2)** **Similar Routine**	Manuela: [During meals at company cafeterias], one watched the other. [We said:] “Remember Tutupapão!” [the mascot used for the campaign]. In my house, I've been doing this since 2015. So, we no longer have a food waste problem; “Put [on your plate] what you can eat. Then, if you think it was too little, you can help yourself again.” No problem!
**47.**	**Manuela** **(FG-2)** **Different Routine**	Manuela: This waste issue is so interesting. For example, in my house, it's just me and my son. So, when I make a cake it's for me and him. On the second day, he says: “Mom, take it to work”. Because he knows that when I get it to the company, people are going to eat it. But if the cake stays at home, it will spoil, because it's just the two of us. So, generally, what I prepare at home I bring here later. It's so normal.
**48.**	**Arthur** **(FG-2)** **Similar Routine**	Arthur: Education involves everything, doesn't it?! [At the company,] they place reminders on the computer [with messages about actions to reduce energy consumption]: when leaving the room, you must turn off the air conditioning, turn off the computer, etc. [...]. So, I think this is the big issue: we have to reduce consumption. And this affects us at home. For example, at home, the light bulbs are burning out. I'm spending [more money] to install LED [bulbs]. [...] The company has already started installing LED lighting in some places. So, this is an issue that also influences us at home.
**49.**	**Alice** **(FG-2)** **Similar Routine**	Alice: I'll speak for myself. I do this analysis all the time about what I'm going to buy; how much packaging I'm buying, how much trash I'm buying, I immediately think about the final destination.
**50.**	**Alice** **(FG-2)** **Similar Routine**	Alice: This issue of using something and [disposing of it, for example], if I take a plastic cup, because I'm away from my office without my mug and I'm going to use a plastic cup, I don't throw that cup away, I have to use it at least twice. So, if I pick up a [disposable] cup to drink water [when I'm out], I keep it in my hand, because I don't have the courage to throw it away. So, I really wanted this feeling to be more than just mine, I wanted it to be a collective thing [...]. Because we get very sad when we see rubbish thrown on the ground, because unfortunately we see it. [...]. But my feeling, my dream is that everyone be this concerned, moving from the individual to the collective. Because I think the company as a whole has this.

**51.**	**Olívia** **(FG-2)** **Different Routine**	Olívia: I also don't have a degree in the environmental field, I have a degree in international relations and I also wasn't aware of this [paying attention to environmental routines]. [...]. But today, I observe it; I wonder how this is such an obvious thing, but I didn't know! And now I have to keep making efforts trying [… to influence] my mother and my stepfather, who are [...] more stubborn people, and I have to keep telling them, for example, to not [consume water to] wet down the road in front of the house. But they think it's great, because the dust will settle down. And you know?! I acquired all of this here. And then, I have hope to be able to change my stepfather and mother's minds. If we can change someone, it's worth it!
**52.**	**Alice** **(FG-2)** **Different Routine**	Alice: My most recent personal experience, and I dare say it is the strongest for my personal life, was the collective strength that this company had to face the water crisis. It was something that shook me very strongly. Because I realized how much better we can still be in a company that already considered itself very good at a certain process [water use and management]. And we discovered that we can be much better, this was the result of the collectivity... And, I confess that I thought to myself like this, if a company this big can do it, why can't we, at home? And reduce our water and electricity bills? Today I tell you, my electricity bill is half of what it was before, my water bill has also dropped a lot. This is due to an internal effort with the small community in my house. [...] The company was a great example for me, it moved me; I thought: "Wow, why can't I do this at home?" [...] This has had a very strong impact on my personal habits.
**53.**	**Alice** **(FG-2)** **Similar Routine**	Alice: Well, I've already taken all this opportunity to carry out actions in favor of the environment, at my home and in other people's homes. Nowadays, I participate in an NGO, where we work in the neighborhood, in the community, together with other NGOs. And this is the result of what we experience here at the company, [...], it involves you so much that you see it as a change in your life, in your actions and you want to spread that. There is also the feeling of belonging, which one of my colleagues mentioned before. So, we feel part of this [at the company]. So, we are unhappy when we see paper on the floor, mixed garbage. We really suffer, we feel sad because we know that this is not the company's culture. We know that this is not what we want. And in my house, since my children were babies, we have been working on this. So, environmental education, selective waste collection, which is the simplest thing and the first thing that everyone thinks of when talking about environmental issues, we implemented not only at home, but also in the building; and [in the community] we helped more than thirty buildings to implement it, together with City Hall. This participation in having initiatives, also, demanding that governments act... I also think that all these actions began here at the company. So, for everything about my life, I took that on.
**54.**	**Vinicius** **(FG-2)** **Similar Routine**	Vinicius: I'll give you an example from my life[...]. Before [I joined the company], my notion of the environment was [limited to] vegetation, forest, sea. [...] Like this issue of waste that we are talking about here, I had no idea. It has changed since I came here [to the company]. From the first moment I entered here […]. With this approach, when I went home, when I got home, I became really different. [...]. Today things are materializing, concerning […] waste [...] Many people figure this out because they work here and are directed towards this. But outside the company, most people never think about it. […] We established waste selective collection, disposal, which before was not available in the building.

**55.**	**Vinicius**<br>**(FG-2)**<br>**Similar Routine**	Vinicius: So, I say, what I received here, in contact here, I took home, took to the condominium where I live. I'm the sub-manager of the condominium. [There,] we set targets for water consumption per apartment, and we signed an agreement to meet this target. […] We implemented many things in the condominium, including reducing costs. And why was this possible? As a consequence of what we had here, taking courses, everything I learned, I learned here, on a daily basis. And I took this to the condominium, to home.
**56.**	**Ronaldo**<br>**(FG-3)**<br>**Different Routine**	Ronaldo: Talking to colleagues here at the company, when I thought about changing home appliances, I went to talk to one of my colleagues. He informed me about the models that have this [inverter] system. It's a new technology, it's about two or three years old.
**57.**	**Matheus**<br>**(FG-3)**<br>**Similar Routine**	Matheus: One thing I learned here at the company was to use a two-liter PET bottle inside the toilet tank. You fill the PET bottle with water and close it, then place it inside the tank. This does not reduce [the efficiency] when flushing the toilet, the pressure comes out the same way...<br><br>Paulo: But does it reduce the volume of water in the tank?<br><br>Matheus: ... with each flush you save two liters of water.<br><br>Interviewer: And did you do this at home?<br><br>Matheus: Yes, I did it at home. I learned about it here, from a work colleague who taught me that.<br><br>Interviewee: I learned this today [now]! I didn't know that! I'll do it at home.
**58.**	**Matheus and Lorenzo**<br>**(FG-3)**<br>**Different Routine**	Matheus: I think that here, everyone at home has a washing machine. I'm pretty sure that everyone reuses the water from the washing machine: at the end of the process, you take a bucket to store the water to wash the laundry area. I took a course [at the company] for six months, and we talked about [...] reusing water, to wash [work] areas, vehicles.<br><br>Lorenzo: I think we are doing our part.
